# Seasonal bacterial niche structures and chemolithoautotrophic ecotypes in a North Atlantic fjord

**DOI:** 10.1038/s41598-022-19165-w

**Published:** 2022-09-12

**Authors:** Eric J. Raes, Jennifer Tolman, Dhwani Desai, Jenni-Marie Ratten, Jackie Zorz, Brent M. Robicheau, Diana Haider, Julie LaRoche

**Affiliations:** 1 Department of Biology, Dalhousie University, Halifax, NS B3H 4R2 Canada; 2 Flourishing Oceans, Minderoo Foundation, Broadway, WA 6009 Australia; 3 Department of Pharmacology, Dalhousie University, Halifax, NS B3H 4R2 Canada; 4 Department of Geoscience, University of Calgary, Calgary, AB T2N 1N4 Canada; 5 Faculty of Computer Science, Dalhousie University, Halifax, NS B3H 4R2 Canada

**Keywords:** Microbial communities, Ecology, Microbiology, Ocean sciences, Marine biology

## Abstract

Quantifying the temporal change of bacterial communities is essential to understanding how both natural and anthropogenic pressures impact the functions of coastal marine ecosystems. Here we use weekly microbial DNA sampling across four years to show that bacterial phyla have distinct seasonal niches, with a richness peak in winter (i.e., an inverse relationship with daylength). Our results suggest that seasonal fluctuations, rather than the kinetic energy or resource hypotheses, dominated the pattern of bacterial diversity. These findings supplement those from global analyses which lack temporal replication and present few data from winter months in polar and temperate regions. Centered log-ratio transformed data provided new insights into the seasonal niche partitioning of conditionally rare phyla, such as Modulibacteria, Verrucomicrobiota, Synergistota, Deinococcota, and Fermentibacterota. These patterns could not be identified using the standard practice of ASV generation followed by rarefaction. Our study provides evidence that five globally relevant ecotypes of chemolithoautotrophic bacteria from the SUP05 lineage comprise a significant functional group with varying seasonal dominance patterns in the Bedford Basin.

## Introduction

Coastal regions and estuaries are the interface between land and ocean, and as biogeochemical hot spots they are responsible for ~ 19% of global oceanic net primary productivity^1,2^. However, along with all biomes on earth, coastal ecosystems are undergoing changes at different temporal scales both naturally and from anthropogenic pressures^3,4^. Measuring temporal change in these ecosystems is an issue of ongoing concern, given the valuable ecological, cultural and economic services they provide^1,5^. Monitoring programs that generate multi-disciplinary time-series datasets have been instrumental in detecting temporal patterns of change and seasonal shifts/cycles, alongside variability in environmental parameters and biological communities^6,7^. Furthermore, high-frequency sampling has allowed quantification of the impacts of short- and long-term stressors on coastal ecosystems specifically^8–10^ and marine ecosystems in general^11,12^.

At the base of the marine food web, pro- and eukaryotic microorganisms sustain, inject and control the fluxes of energy and nutrients, and provide the organic matter for all higher trophic levels^13^. Despite considerable data gaps in certain coastal areas (e.g., the Atlantic South American coast and most of the Indian Ocean), the continued international effort in managing coastal time-series is delivering valuable mechanistic insights into the seasonal trends, periodicity, and phenology of marine prokaryotes^9,14–20^. This concerted effort highlights the need to identify seasonal patterns and changes in prokaryotic diversity as these will provide baselines to disturbances and long-term trends. Furthermore, while marine prokaryotes respond to external changes, they also play a crucial role in shaping their environment through the microbial loop^21^; any changes in their community structure could thus have important ramifications for biota higher up the food chain^22,23^.

In this study we present findings from a 4-year time series of bacterial *16S* ribosomal RNA gene (rRNA) metabarcoding data in Bedford Basin, a temperate coastal embayment in the Northwest Atlantic Ocean. Samples were collected weekly from four depths congruent with physical and biochemical metadata. Traditionally, monitoring occurs at monthly or quarterly intervals, which may be insufficient to capture important transitions and disruptions to the community such as transient bloom periods or severe weather events (i.e., shorter-lived community dynamics^24,25^). The goal of this study was therefore to use high-resolution weekly sampling to describe the yearly reoccurring cycles, and hence in situ dynamics, of prokaryotic communities, and to describe important metabolic pathways. First, we calculated taxonomic diversity indices across time to test whether day length, rather than temperature and nutrients alone, was the environmental variable most effectively describing changes in prokaryotic alpha and beta diversities over an annual cycle. Next, we aimed to identify whether there were marked seasonal changes in abundance for different phyla, and if these results could be further supported by metabolic pathways inferred from PICRUSt2 analyses of *16S* rRNA gene sequences^26^. Finally, we tested whether different ecotypes within one of the diverse prokaryotic clades observed at our site exhibited seasonality over an annual cycle^27^. In this regard, we investigated the chemolithoautotrophic bacterial SUP05 clade, which comprise a significant functional group in suboxic (< 100 µmol O_2_ L^−1^) dark (aphotic) coastal waters^28^, such as the Bedford Basin.

## Results and discussion

### Seasonal changes in a North Atlantic embayment

**Figure 1:**
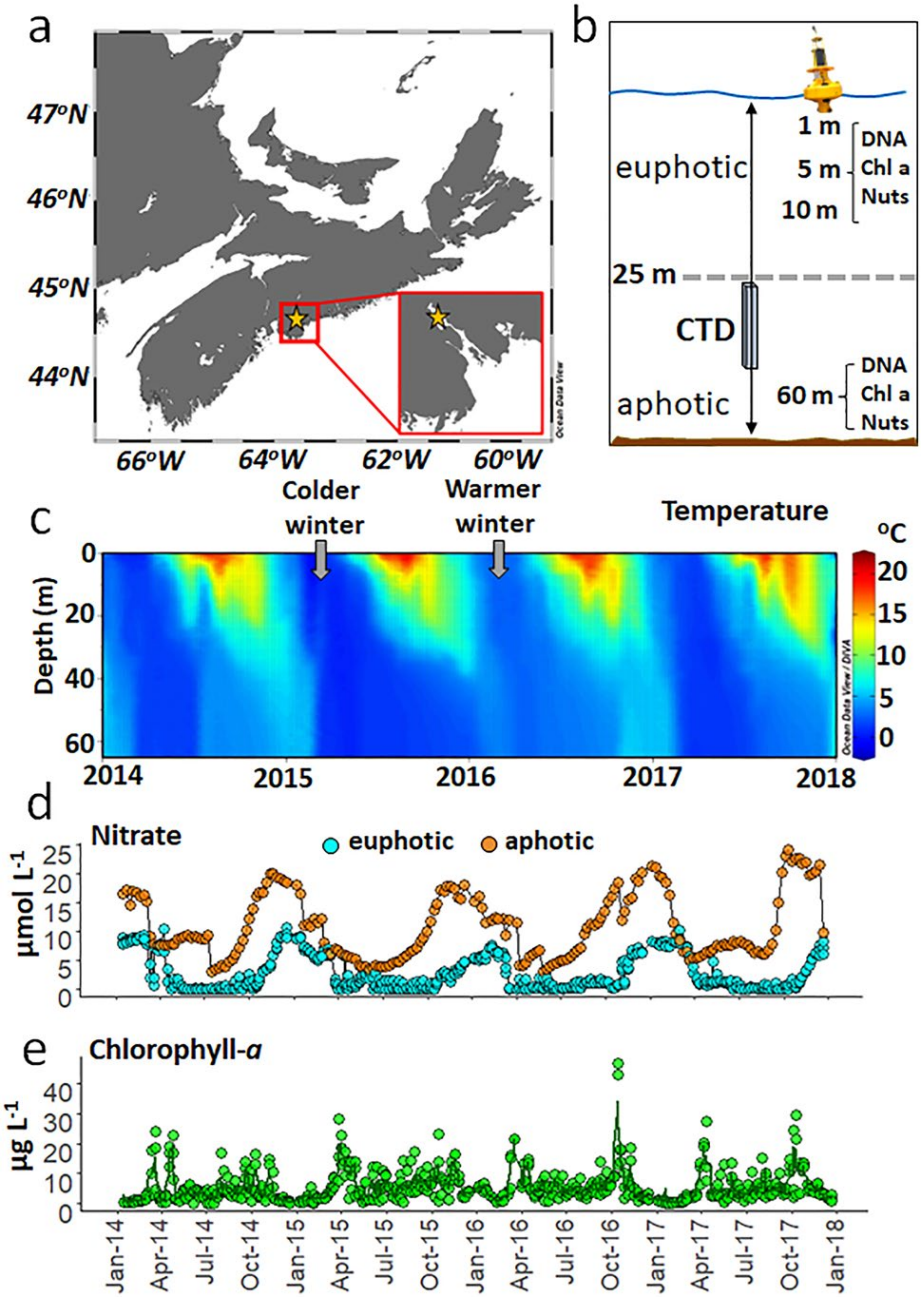
Our sampling site was located in Nova Scotia, Canada (**a**), and specifically within the Bedford Basin [inset of **a**; sampling site (star) is also known as HL0 = 44.6936 latitude, −63.6403 longitude]. (**b**) Schematic representation of variables and conditions measured during this study, included: sampling depths for DNA, chlorophyll-*a* and concentrations for dissolved nutrients (nuts; which included PO_4_^−^, NO_3_^−^, NO_2_^−^, NH_4_^+^ and Si) along with CTD profiles. Weekly measurements from 2014 to 2017 included: temperature profiles (**c**), nitrate concentrations in the euphotic (1, 5 and 10 m; cyan circles) and in the aphotic zone (60 m; orange circles; **d**), and chlorophyll-*a* concentrations in the euphotic zone (the green line is the average for the three depths 1, 5 and 10 m; **e**). Grey arrows denote colder and warmer winters for 2015 and 2016, respectively, as detailed by Haas et al.^32^. Image credit for yellow buoy; Robert Brazzell.

The Bedford Basin is a coastal embayment with a maximum depth of 71 m and is connected via a narrow (500 m) and shallow (20 m) sill to the Scotian Shelf in the northwest Atlantic (NWA; Fig. 1a,b). Lower density surface waters flow out to the NWA, while deeper, slightly more saline waters flow into the basin over the sill^29^. Lowest salinity values during our study (25.40) were measured at the surface, and highest values (31.58) near the bottom. The average water column salinity was 30.09 ± 0.88 and was not significantly different between years from 2014 to 2017 (Wilcoxon tests *p* > 0.1; Supplementary Table 1). The euphotic depth is shallower than 30 m year-round, and like the Scotian Shelf, the basin is subject to distinct temperate seasonal cycles^30^ (Fig. 1d). As is typical of other northern temperate fjords, the Bedford Basin also experiences seasonally defined water column stratification accompanied by spring (~ April) and autumn (~ September) phytoplankton blooms (Li^31^ and Fig. 1c,e). This seasonal cycling also influences the available absolute macronutrient (N and P) concentrations as well as their stoichiometry, with a depletion of N relative to P in summer (Supplementary Fig. 1). Furthermore, the suboxic conditions that develop in the deep water during late autumn (< 100 µmol O_2_ L^−1^; below 30 m and Supplementary Fig. 2) are generally dissipated by the convective mixing of the entire water column that results from colder temperatures, replenishing dissolved oxygen in the deep water and nutrients in the surface waters.

### Alpha diversity peaks in winter and Beta diversity follows a resilient multi-annual cycle

**Figure 2:**
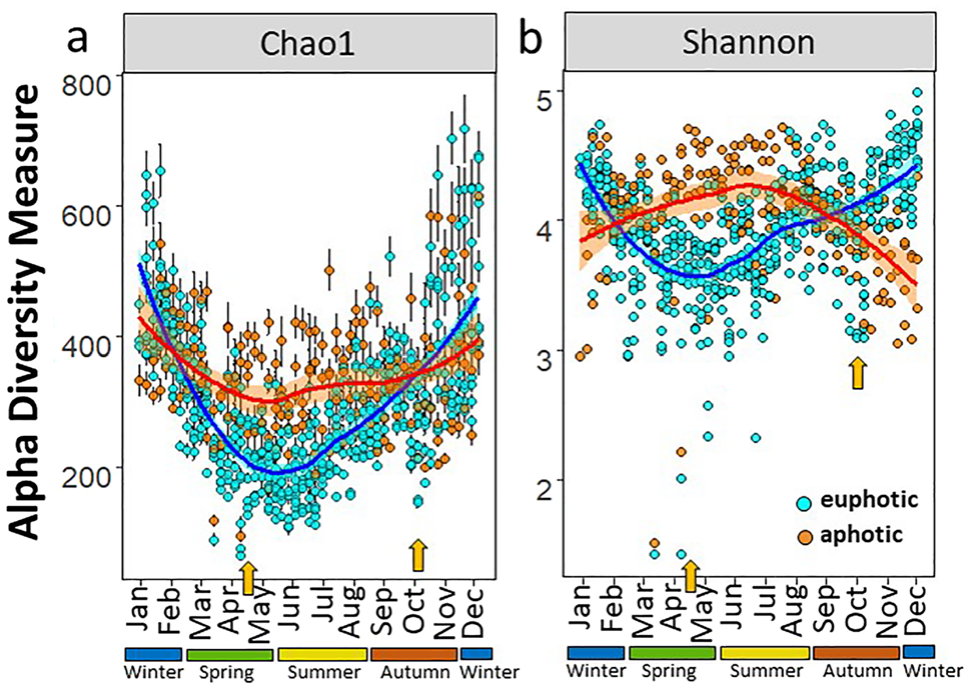
Seasonal trends for alpha diversity indices in the Bedford Basin from 2014 to 2017. Chao1 diversity (**a**; with standard errors as defined for the Chao1 model for estimating richness), and Shannon diversity (**b**). Orange arrows indicate low alpha diversity during the spring and autumn blooms. Samples from the euphotic (1, 5 and 10 m; cyan circles) and aphotic zone (60 m; orange circles) are colour coded. Loess regression lines are fitted for each zone and the inflection points on the regression lines show a minimum in April/May and in October for both indices.

Across the four years, both Chao1 (species richness) and Shannon (evenness) diversity indices for prokaryotes showed a clear seasonal trend in the upper 10 m of the water column, with higher diversities in the colder, nutrient-rich, winter months (Fig. 2). Lowest alpha diversity was found during early summer (Chao1) and the productive spring bloom (Shannon; Fig. 2). Changes in alpha diversity at 60 m depth, although still statistically significant, were less pronounced compared to the surface waters (quadratic fits in the aphotic zone had lower R^2^; see Supplementary Table 2 and Supplementary Fig. 3). This aligns with the previously observed lack of global diversity gradient in the aphotic pelagic zone^33^. The weak seasonal trend in the aphotic waters could, in our study, be best explained by an increased stability of the deep water compared to the surface; i.e., smaller seasonal variations in temperature and minimal in situ autotrophic production which have been suggested to be the two main variables driving changes in richness trends (i.e., the kinetic energy^33^ and resource hypotheses^34^). Possible explanations for the high diversity in the euphotic zone during the colder winter months are (1) mixing of the water column will introduce species to the surface that usually reside at depth^32^ and (2) an increased range of available nutrients (i.e., NO_3_^−^, PO_4_^3−^ and Si) in the euphotic waters will support more ecological niches and therefore more species (resource heterogeneity). The lower diversity during the bloom periods could be related to the proportional increase of fast growing specialists such as Flavobacteria, which thrive in coastal marine waters during phytoplankton blooms^35^. Their temporal niche advantage is supported by their ability to express specific degradation and transport genes for algal-derived (exo)-polysaccharides^36^. However, further metabolic rate measurements are needed to test whether the lower prokaryotic diversity during the productive months leads to a lower functional diversity^37^.

Across the 4-year study, our results highlight a seasonal reversal in prokaryotic diversity in the upper water column relative to productive and warmer waters in summer. Similar peaks in diversity in winter have been observed in the temperate latitudes at a regional scale in the English Channel^24^, in polar regions such as the Fram strait^38^ and on a global scale^39^. The seasonal patterns observed in our data remained present at higher rarefaction depths (to 20,000 reads) confirming the robustness of these trends (Supplementary Fig. 3). All seasonal comparisons in the euphotic zone, with the exception of spring and summer for Chao1 and autumn and winter for the Shannon index, were significantly different from each other (Wilcoxon tests *p* < 0.05; Supplementary Tables 3–4). Similar seasonal trends also remained regardless of rarefaction depths for the rare microbiome (prokaryotes which comprised < 1% of the community; Supplementary Fig. 4).

**Figure 3:**
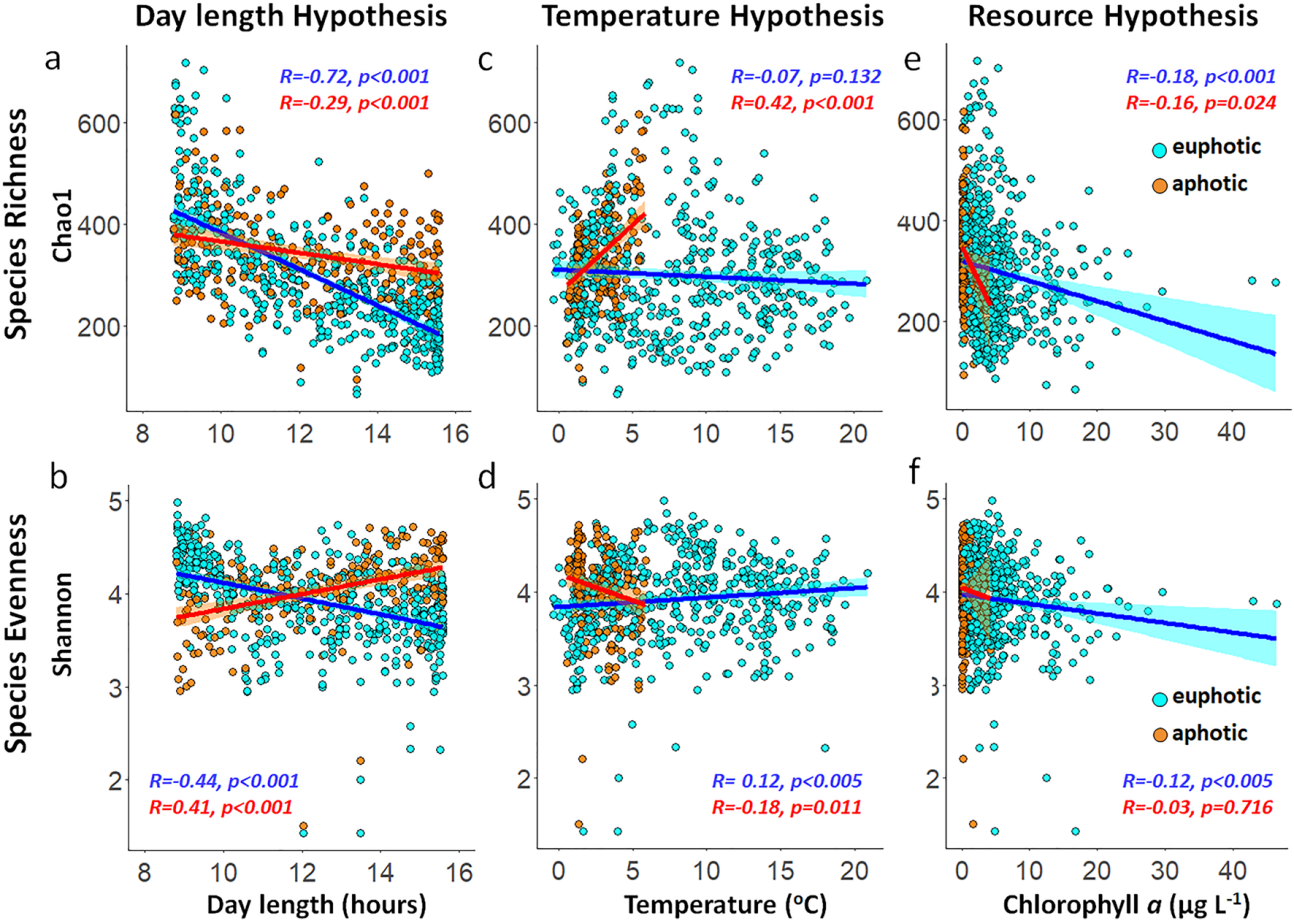
Alpha diversity trends in the Bedford Basin from 2014 to 2017. Species richness (Chao1) and species evenness (Shannon diversity) were correlated with day length (**a,b**), temperature (**c,d**), and chl-*a* concentrations as a proxy for biomass production (**e,f**). Linear regression lines are shown in blue for the euphotic zone and in red for the aphotic zone. Pearson correlation coefficients are indicated as R on the plots with their associated *p*-value. The ASV table was rarefied to 5000 reads prior to analyses, and seasonal patterns remained present independent of rarefaction depths (Supplementary Fig. 3).

Spatial patterns in prokaryotic richness and diversity have mainly been explained by two hypotheses: the kinetic energy hypothesis and the resource hypothesis. The former postulates that higher temperatures increase the metabolic rate of pathways, resulting in higher speciation rates and ultimately in higher alpha diversities (Sunagawa et al.^40^ and Ibarbalz et al.^33^, and reviewed by Brown^41^). The latter suggests that more energy production will support a higher numbers of species^34,42^. The observation that day length may overrule the kinetic energy hypothesis (Fig. 3) is in contrast to the findings from a number of studies showing that temperature appears to be the strongest positive predictor of prokaryotic diversity in the global ocean (i.e., see Fuhrman et al.^43^, Ibarbalz et al.^33^ and Mittelbach et al.^34^). Furthermore, the seasonal trends in our time-series data do not support the productivity/resource hypothesis which show that autotrophic productivity is positively correlated with prokaryotic richness (i.e., see Mittelbach et al.^34^ and Raes et al.^42,44^; and Fig. 3). Instead, day length had the strongest correlation coefficients for both species richness and evenness in the euphotic zone (Fig. 3). Multiple regression models (with temperature, chl-*a* and day length fitted last) also supported these findings and showed that day length contributed the largest proportion to the models in the euphotic zone (Supplementary Fig. 5). For completion we note that the richness peak between 5° and 10 °C in the euphotic and aphotic zone (Fig. 3c,d) coincided with the winter months (Supplementary Fig. 6).

**Figure 4:**
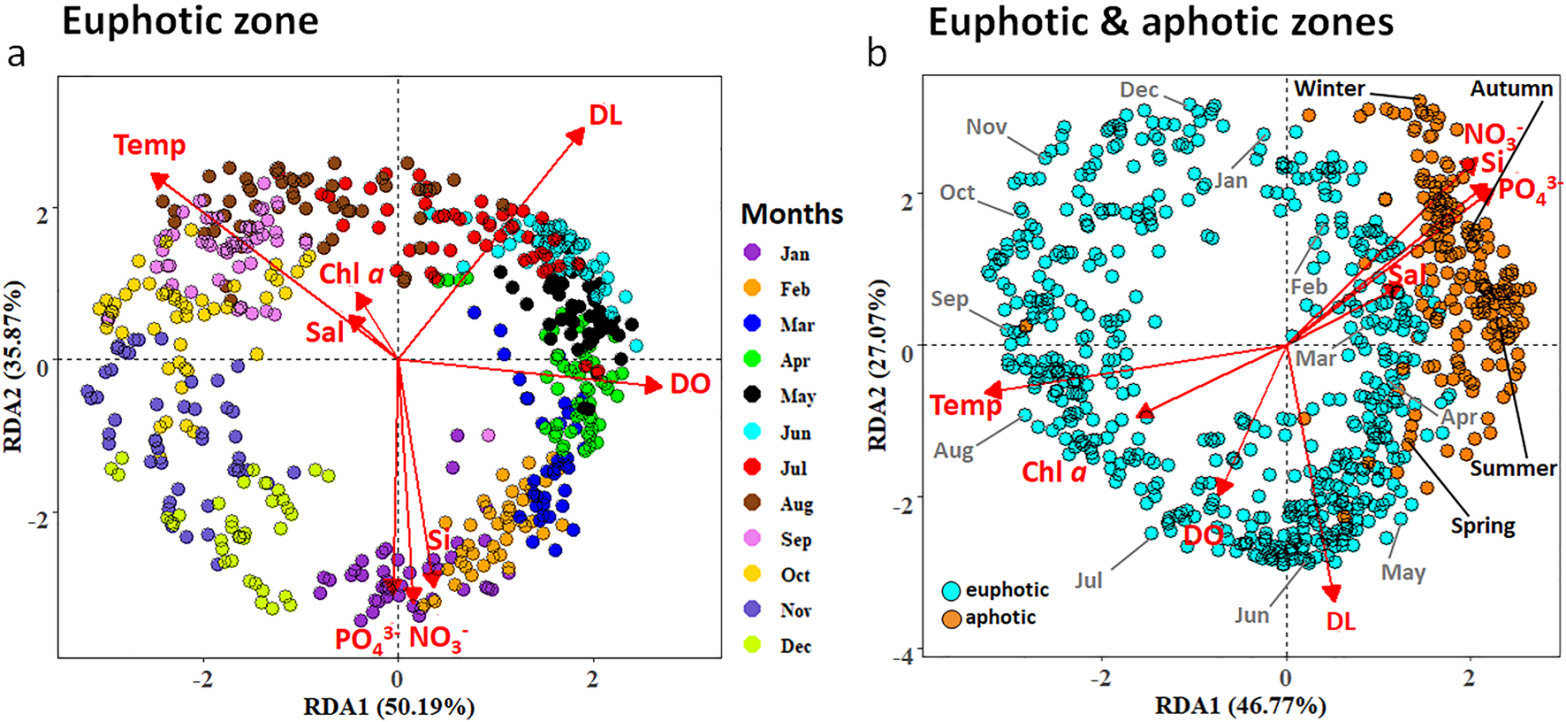
Redundancy analyses (RDA) to characterize the specific abiotic variables in the water column which exerted the largest influence on the prokaryotic community composition in euphotic zone only (**a**) and across all four depths (**b**). The ASV data were CLR transformed and environmental parameters were standardized with the ‘standardize’ function using decostand from the Vegan Package^45^. Months are coded with different colours (**a**) and sampling depths are colour coded in cyan (euphotic zone; 1, 5, 10 m) and orange (aphotic zone; 60 m; **b**). Abbreviated months are shown in grey, and seasons are highlighted in black for the aphotic zone (**b**). Vector abbreviations are temperature (temp), salinity (sal), chl *a* (chlorophyll-*a*), dissolved oxygen (DO), day length (DL), nitrate (NO_3_^−^), silicate (Si), phosphate (PO_4_^3−^).

Redundancy analyses (RDAs) confirmed that day length had the strongest correlative power to explain variance in the prokaryotic community, similar to the results from Gilbert et al.^24^ for the L4 time series. In our study, day length alone explained 91% of the variance in the prokaryotic community (Supplementary Table 5). Overall, regression fitting of single explanatory variables including temperature, NO_3_^−^, PO_4_^−^, Si and DO were also all significantly correlated with the prokaryotic community composition (r^2^: 0.83, 0.71, 0.67, 0.62, 0.51 respectively; all *p* < 0.001; Supplementary Table 5). Both chl-*a* concentrations and salinity also revealed significant correlations with the prokaryotic community composition, but each parameter contributed < 8% of the variance (r^2^: 0.07, 0.04 respectively; all *p* < 0.001; Supplementary Table 5). RDA showed that beta diversity was largely explained by changes in day length (similar to alpha diversity), and that seasonality again seemed to overrule both the kinetic energy and the productivity/resource hypotheses. Partial Mantel tests were used in an attempt to disentangle the effects of day length and temperature on the prokaryotic beta diversity. The Mantel correlation statistics between beta diversity and temperature, controlled by day length (*r*: 0.494, p < 0.001), and between beta diversity and day length, controlled by temperature (*r*: 0.437, p < 0.001) were very similar. These results corroborate the findings from our RDA analyses where both temperature and day length revealed highly similar r^2^ values (Fig. 4a and Supplementary Table 5). Day length and temperature covary and the results from both the Mantel test and RDA analyses highlight the difficulty of disentangling these factors. While alpha diversity revealed a significant negative relationship with day length, the main driver for the beta-diversity pattern remains unresolved.

The constrained (RDA; Fig. 4a) and unconstrained (PCA; Supplementary Fig. 7) prokaryotic community analyses in the euphotic zone displayed a clear seasonal pattern, and no significant differences were found within the euphotic zone (ANOSIM, all *p*-values > 0.05 between samples from 1, 5 and 10 m, nested within sampling month, across all four years; see Supplementary Table 6). Samples from the aphotic zone (60 m), however, were significantly different from the euphotic zone (ANOSIM *p* < 0.05; see Supplementary Table 6). The communities in the aphotic and euphotic zones were more similar in winter, when a well-mixed water column (Fig. 1c) reduced the proportion of unique ASVs in the aphotic zone by half compared to summer (Supplementary Fig. 8). The increase in NH_4_^+^ concentrations in the aphotic zone from spring to autumn suggest the remineralisation of organic matter (Supplementary Fig. 2b and Haas et al.^32^). The export of the particle attached surface prokaryotic community to the deeper waters during the productive months may contribute to higher alpha diversities in summer in the aphotic zone. Sinking particles and faecal pellets are known to vertically connect prokaryotic communities in the water column^46^, and suggest that sinking particle-attached bacteria can continue to degrade the exported organic matter in the aphotic zone.

Monthly differences, within a sampling year were significantly greater than interannual differences (i.e., in the euphotic zone ANOSIM Global R-values averaged 0.796 ± 0.045 (± sd) and Fig. 4a) between months across all years, while the interannual Global R-value was 0.091; Supplementary Table 7 and 8). The lower global R-values between years provide evidence that the seasonal cycle was consistent multi-annually (a resilient seasonal cycle; Fig. 4). These findings of a resilient seasonal cycle reaffirm the strong cyclic patterns previously recorded in the Bedford Basin by El-Swais et al.^47^, who investigated prokaryotic seasonality by targeting the V5 region of the *16S* rRNA gene (100 bp) from one millilitre of formalin‐fixed seawater using 45 samples spanning six years (2005–2010). Our study, however, used weekly samples (*n* = 792) across four years, and the *16S* rRNA gene V4-V5 region (412 bp) was amplified from 500 ml of filtered seawater, with sequences clustered at an ASV level rather than a 90% identity cut‐off as done by El-Swais et al.^47^. The longer amplicon sequence combined with the ASV analyses enabled a more in-depth description of the temporal changes in the prokaryotic community and improved taxonomic assignment. Clear examples are the higher variability in winter (probably due to a higher alpha diversity or an artefact due to the lower concentrations of particulate organic matter (biomass) relative to spring and autumn; Supplementary Fig. 9), and the findings of temporal trends of conditionally rare taxa and ecotypes as shown below.

As discussed in Haas et al.^32^, notable events across our 4-years of weekly observations included: (i) a significantly colder winter period in 2015 (Wilcoxon test *p* < 0.05); with lowest water temperatures measuring -0.35 °C in the upper 10 m (Fig. 1c), resulting in stronger winter mixing relative to the other years; (ii) higher minimum temperatures in the winter of 2016 (1.51 °C in the upper 10 m), resulting in reduced winter mixing relative to the other years (Fig. 1c); and (iii) short-lived (~ 3 to 4 weeks) intrusion events detected as relatively higher oxygenated waters which interrupt the stratified period (below 30 m; Supplementary Fig. 2). While these intrusions can impact the N-cycling fluxes mediated by ammonium-oxidising microorganisms in the deeper waters, they do not impact the vertical stratification of the water column^32^. Although we were able to detect these events as significant environmental disturbances, the recurring beta diversity patterns (Fig. 4) suggest that neither the intrusion events, nor aberrant colder winter temperatures impacted the prokaryotic community assemblages.

In summary, our results and those of others^18,24^ support the premise that seasonal fluctuations dominate the pattern of prokaryotic diversity in temperate regions^39^. Although global ocean surveys (e.g., TARA Oceans expeditions) have observed that highest prokaryotic diversity occurs near equatorial latitudes^33^, the lack of seasonal observations, compounded by the difficulties of sampling during winter months in polar and temperate regions, bring into question our current perception of global microbial diversity patterns. Recent studies utilizing time-series data have shown that species richness is negatively correlated with day length over an annual cycle with a highest richness peak in winter^24,38^. The finding that the relationship between temperature and diversity changes from a regional to a global scale re-emphasises the need to adjust our sampling designs and analyses by seasons if we want to truly understand the drivers of marine prokaryotic richness and its influence on the metabolic potential of the community^48^. These findings are especially important for temperate and high latitude regions which are undersampled in winter, whereas tropical and equatorial oceanic zones with modest variations in day length are consequently expected to be less impacted by seasonal change.

### Seasonal niche partitioning of bacterial phyla and functional pathways

**Figure 5:**
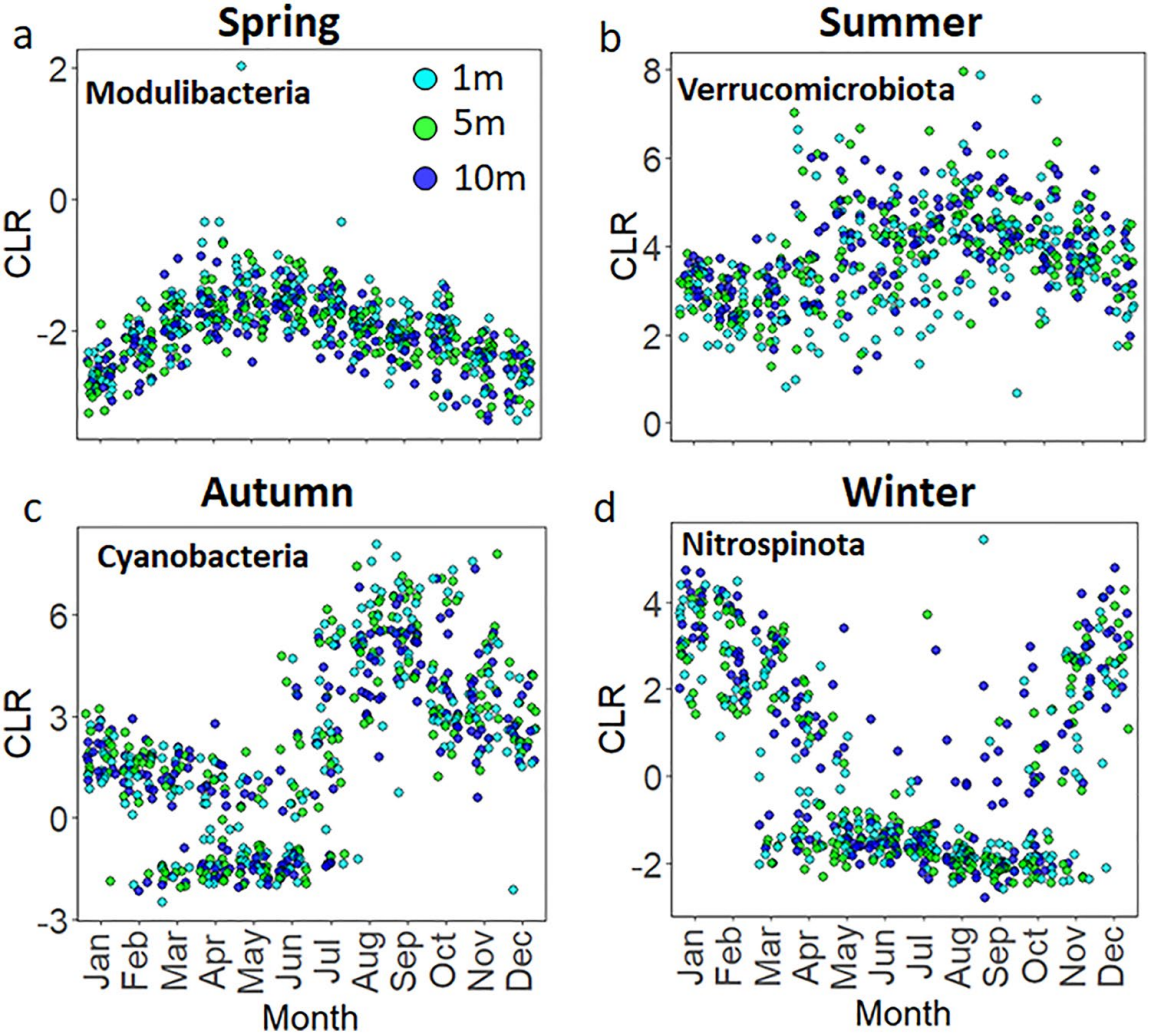
Seasonal changes in bacterial phyla in the euphotic zone (1, 5 and 10 m) in Bedford Basin. Significance patterns and temporal niches were assessed with a permutation test (“multipatt” function from the Indicspecies package^52^; see Methods and Supplementary Table 9). Due to the compositional nature of the sequence data, the changes are visualized with CLR values rather than relative abundances.

The increased recognition of the compositional nature of microbiome datasets^49^ encouraged us to use the centred log-ratio (CLR) transformation to explore relative changes and seasonal trends in the prokaryotic community. Using CLR transformed data, we identified several bacterial phyla that have temporally defined niches in the euphotic and aphotic zones (Fig. 5; Supplementary Fig. 10 and Supplementary Tables 9 and 10). We also present the change in relative abundances of certain bacterial genera using rarefied data in contrast to the CLR transformed data (Supplementary Fig. 11). While the main seasonal trends remain the same for the abundant bacterial phyla (i.e., similar trends are seen for the Cyanobacteria and Nitrospinota in autumn and winter respectively when the data are rarefied or CLR transformed; Supplementary Fig. 11), the CLR transformed data revealed new insights into the seasonal niche partitioning of phyla with low relative abundances, thereby adding a new level of resolution for rarer taxa previously unavailable^47^. Clear examples, in spring and summer, were revealed for the bacterial phyla Modulibacteria and Verrucomicrobiota (Fig. 5), and Synergistota, Deinococcota, and Fermentibacterota (Supplementary Table 9). However, when the ASV table was rarefied and expressed as relative abundances, the above phyla (which contributed < 0.1% of the relative bacterial abundance) either did not show any seasonal trends or were removed during rarefaction (Supplementary Fig. 12). Bacteria present in low relative abundance can play significant roles in the C and N-cycles; Musat et al.^50^, for example, demonstrated that an anaerobic phototrophic bacteria representing 0.3% of the community contributed a large fraction of the total C-fixation and NH_4_^+^ assimilation rates (up to 70 and 40% respectively). CLR transformed data revealed the cyclic distribution pattern of conditionally rare taxa (or “microbial dark matter”^51^) that may be seasonally important in the Bedford Basin, although their specific roles in biogeochemical cycling would require additional studies.

**Figure 6:**
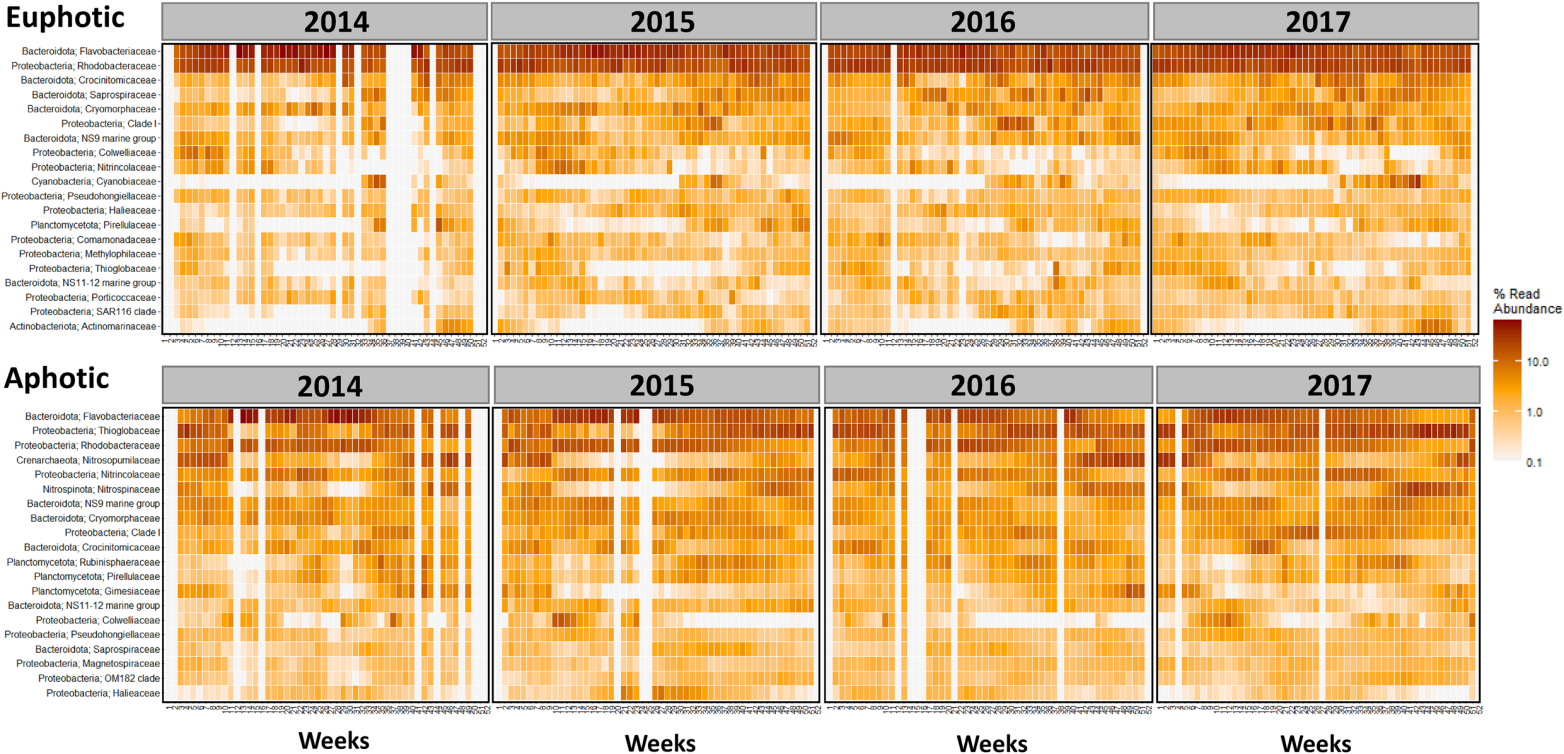
Temporal change in the Bedford Basin. Top panel shows the euphotic zone and the bottom panel the aphotic zone. ASVs were aggregated at a Family level and the Phylum level is added in front. The top 20 abundant taxa are shown and the colour scale is the % read abundance on a log10 scale. Note: Samples with a read depth < 5000 were removed, resulting in empty columns.

A detailed overview of seasonal trends of the top 20 most abundant taxa is shown in Fig. 6. Flavobacteriaceae and Rhodobacteraceae clearly dominated the euphotic zone throughout the year in terms of relative abundances. Crocinitomicaceae and Saprospiraceae revealed higher abundance in winter, whereas the long absences preceding seasonal blooms in Cyanobiaceae, Pirellulaceae, Thiogglobaceae, SAR116 clade and Actinomarinaceae are noteworthy to mention (Fig. 6). In general, our results broadly mirror previous studies^47^ that showed the association of the Flavobacteria such as *Polaribacter* and *Ulvibacter* with the spring bloom (Supplementary Fig. 13), and ammonia-oxidizing species as indicator ASVs in winter months^32^. A similar seasonal succession from Flavobacteria in spring to nitrifying organisms in winter has recently been shown in the Arctic and subarctic regions of the Atlantic Ocean^38^. Diminishing ice cover and the advection of warmer Atlantic water (referred to as Atlantification) have been hypothesised to trigger a shift in phytoplankton diversity and a proportional increase in Flavobacteria at high latitudes. Prolonged summer stratification and reduced wintertime convection due to Atlantification have been further shown to weaken deep-water renewal resulting in a decrease in associated “microbial recyclers”, such as nitrifying Archaea and Bacteria in the Arctic^38^. These microbial recyclers play an important role in the global N-cycle, and in the Bedford Basin, winter mixing was identified as a major variable controlling the abundance and diversity of nitrifying organisms^32^. The seasonal changes in photoautotrophic production (with low alpha diversity) and heterotrophic recycling (with high alpha diversity) periods in our study complement the observations from the Arctic Ocean^38^. The interconnection between nutrient depletion and autotrophic biomass production in spring, and nutrient recycling and renewal in winter is a natural cycle which can be impacted by increasing ocean temperatures which prolong the stratification of the mixed layer^53^. Time-series data, such as those used in this study, will allow a better characterization of how a warmer ocean will impact the biogeochemical fluxes mediated by micro-organisms in the North Atlantic but also in Arctic and subarctic regions, where similar seasonal trends have been observed^38^.

**Figure 7:**
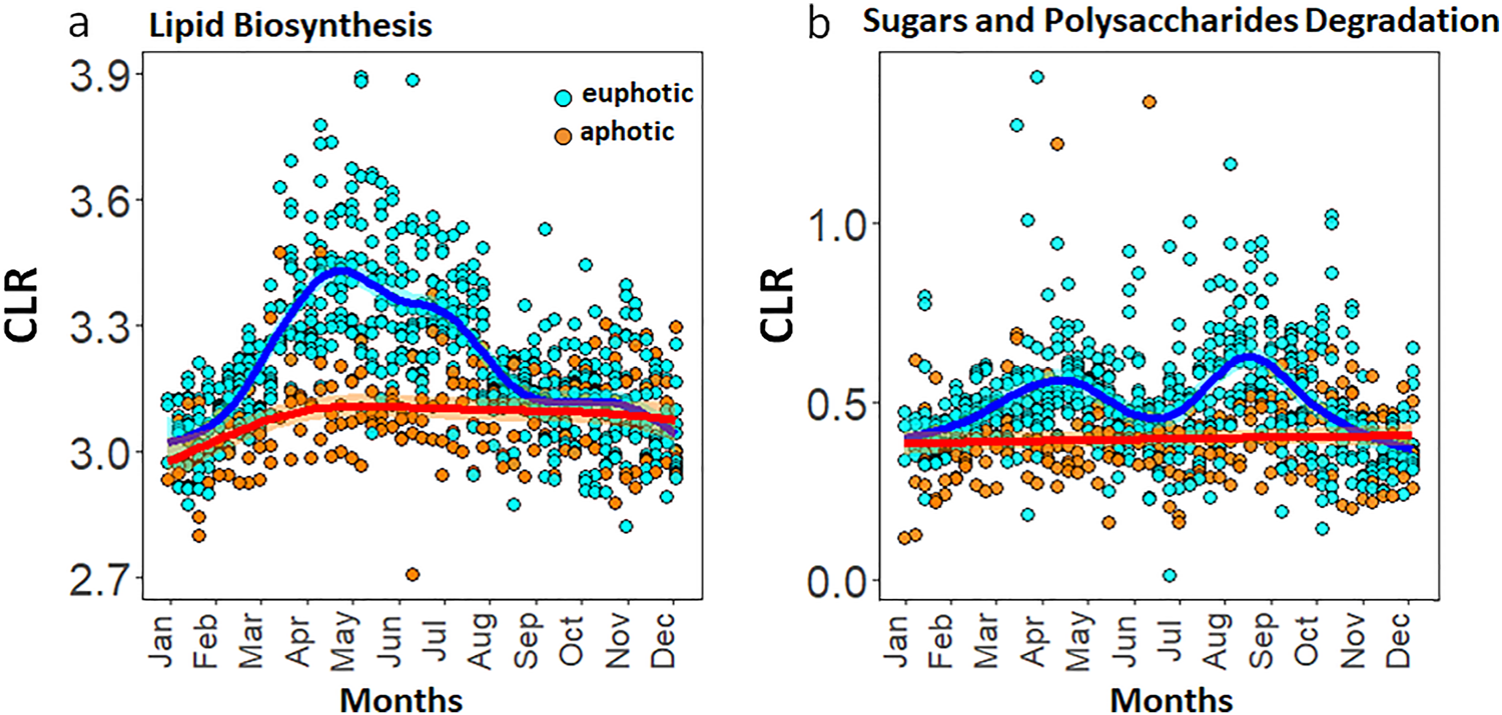
Seasonal trends in lipid biosynthesis pathways (associated with the spring bloom) and Sugars and Polysaccharides degradation (associated with the spring and autumn blooms) in the Bedford basin from 2014 to 2017. Temporal pathways were identified using a permutation test (“multipatt” function from the Indicspecies package^52^; see “Methods” and Supplementary Table 12). Generalized additive mode smoothings are shown in blue and red (**a,b**).

Although the results from metabolic inference-based prediction tools such as PICRUSt2^26^ should be cautiously interpreted^54^, the approach has provided valuable mechanistic insights into the large-scale functional ecology of microbes in open oceanic ecosystems^48^. Here we used inferred metabolic predictions to show the positive correlation between seasonal spring blooms and prokaryotic lipid biosynthesis (Fig. 7a). Polysaccharide degradation pathways showed a positive correlation with both the spring and autumn blooms in the photic zone across four years (Fig. 7b). A decreasing trend with depth was noted with the highest number of predicted lipid biosynthesis and polysaccharide degradation pathways in the surface waters.

The positive relationships between lipid biosynthesis, the breakdown of organic matter, and the spring bloom offer the opportunity to track energy production in the Bedford Basin, and potentially other coastal embayments. Our results show that community-level metabolic data can be used as a qualitative tool for understanding changes in ecosystem functions. These insights, along with the temporal connectivity patterns of pro- and eukaryotic organisms, will allow us to generate new hypotheses regarding how local environmental perturbations, whether natural or anthropogenic, could impact the top-down and bottom-up controls in marine food webs^55^. This will open the possibility to test how changes at the lower microbially-driven trophic levels can propagate to the higher trophic levels and, how taxonomic changes could impact the fluxes of macro- and micronutrients mediated by the prokaryotic community.

### Chemolithoautotrophy and seasonal ecotypes

**Figure 8:**
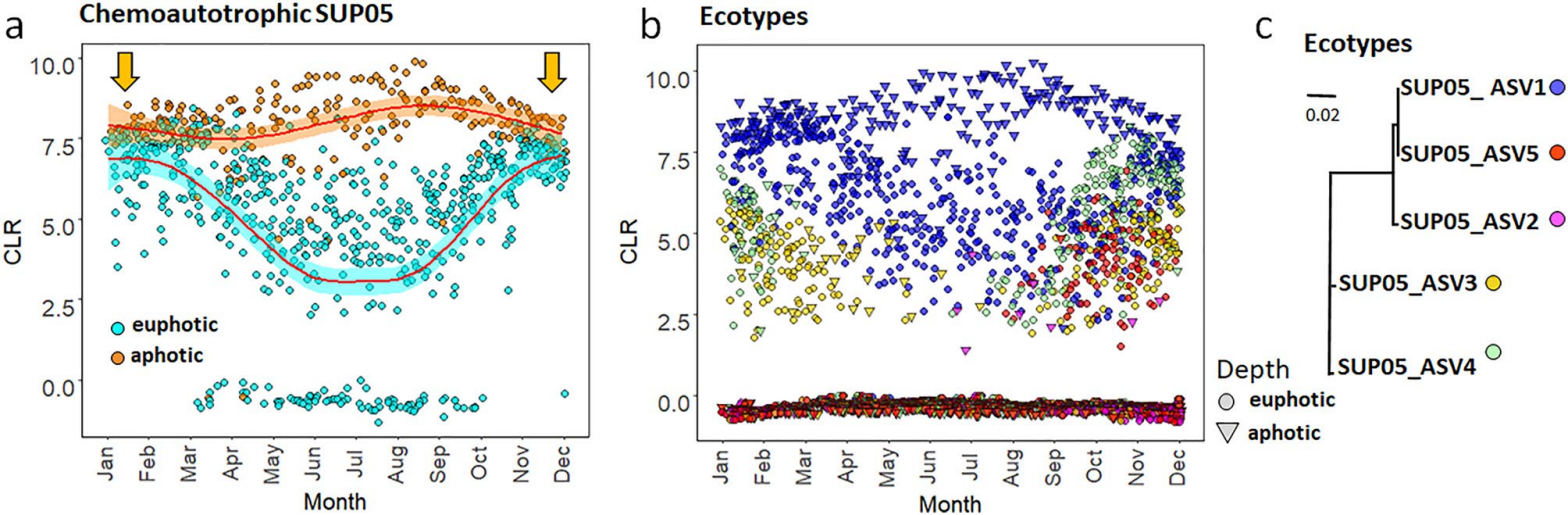
Seasonal trends for the chemoautotrophic SUP05 lineage in the Bedford Basin from 2014–2017. The SUP05 reads are conglomerated at a genus level (**a**) to highlight the differences between the euphotic and aphotic zones, while (**b)** highlights the different SUP05 ecotypes from non-conglomerated reads. Winter mixing occurs during Nov-Feb shown by orange arrows on (**a**). Generalized additive mode smoothings are shown in red (**a**), note: analysis of CLR data reveals how ASVs behave relative to the per-sample average. Sampling depths are presented by different symbols on (**b**), and ecotypes are shown in different colours. The red arrow (**b**) highlights the temporal niche for ecotype SUP05_ASV5**.** Neighbor-Joining tree with a Tamura-Nei genetic distance model for the five SUP05 ecotypes (**c**).

The majority of the metabolic predictions including lipid biosynthesis and polysaccharides-degradation pathways revealed the clearest change across seasons in the euphotic zone. While metabolic pathways also showed seasonal trends in the aphotic zone (Supplementary Table 13), we note two examples which illustrate the limitations in using PICRUSt2 to infer the metabolic potential of marine microbial communities. The first is the absence of nitrification pathways, which have been shown to be important in the Bedford Basin^32^, and the second is the absence of chemolithoautotrophy pathways. In the Bedford Basin, we noted a significant increase in the relative abundance of the chemolithoautotrophic SUP05 clade in the euphotic zone in winter (Fig. 8a), while overall highest relative abundances were observed throughout the year in the deeper suboxic waters of the basin in winter (up to 25% of the relative bacterial abundance). Members from the SUP05 clade are known chemolithoautotrophs and execute important roles in energy production in marine ecosystems including suboxic fjords^28^, deep ocean hydrothermal plumes^56,57^, and coastal shelf systems such as those off the West coast of Africa^58^.

The SUP05 clade of the Bedford Basin contained five distinct ecotypes (ASVs). Three out of the five ecotypes differed from one another by a single nucleotide within the *16S* V4-V5 region (i.e., SUP05_ASV1, SUP05_ASV2 and SUP05_ASV5). SUP05_ASV3 and SUP05_ASV4 also differed from one another by only one *16S* V4-V5 nucleotide, but showed a difference of up to 18 nucleotides for the same gene region with the annually-persistent SUP05_ASV1 (Fig. 8b-c and Supplementary File 1). One ecotype (SUP05_ASV5) showed a clear temporal niche in autumn and two ecotypes (SUP05_ASV3 and SUP05_ASV4) occupied a niche in winter (Fig. 8b). The persistence of SUP05_ASV1 through the spring and summer months could suggest that this ecotype is a mixotroph and competes with phytoplankton for light and nutrients. The potential for autotrophy in the aphotic zone is likely, as members of the SUP05 clade are known to contain large RuBisCo subunit genes (rbcL; a key enzyme during C-fixation; Swan et al.^59^). The co-occurrence of dissimilatory sulfur oxidation (adenosine-5′-phosphosulfate reductase alpha (*aprA*), reverse dissimilatory sulfite reductase (*rdsrA*) and autotrophic carbon fixation (RuBisCO) genes in members of the SUP05 clade suggest that these bacteria oxidise sulfur to support the autotrophic fixation of C^59^. In the Bedford Basin, Taguchi & Platt^60^ showed that annual dark C-fixation in the whole water column contributes up to 25% (50 g C m^−2^ year^−1^) of the estimated annual production, with highest dark C-fixation rates in winter^61^. We propose that the chemolithoautotrophic bacteria from the SUP05 lineage might be a prime candidate for the dark C-fixation rates measured 44 years ago by Taguchi & Platt^60^.

The metabolic flexibility of the SUP05 clade and the persistence of the SUP05_ASV1 ecotype specifically in summer could also be due to (1) an increased ability to use a larger range of reduced organic and inorganic sulfur at sub-micromolar concentrations, and/or (2) the ability to store a larger proportion of intracellular sulfur when it respires oxygen^62,63^. While SUP05_ASV1 remained the dominant ecotype throughout the year, SUP05_ASV3 and SUP05_ASV4 appeared to benefit from the reduction of light and oxygen^32^ during the colder autumn and winter months. This temporal niche separation highlights that the SUP05 ecotypes in the Bedford Basin have adapted specific metabolic variations^27^ which allow them to compete and/or coexist with their close relatives, as has been seen for the Pelagibacter SAR11 and the cyanobacteria *Prochlorococcus* in the open ocean^64,65^. Overall, these findings reaffirm the importance for considering dark C-fixation and sulfur cycling pathways during winter in coastal embayments in the NWA.

## Conclusions

Weekly metabarcoding sampling across four years revealed clear temporal niche structures with a richness peak in winter. Our results from a time series at a mid to high latitude suggest that seasonal variations, integrated here in a deterministic daylength parameter, had a dominant effect on shaping the pattern of bacterial diversity, overriding the contribution of kinetic energy or available resources. Our findings indicate that in temperate to high latitude regions, the measured diversity must be interpreted in the context of an increasingly strong seasonal cycle at high latitude where highest diversity is observed at the shortest daylength period. Day length as a variable is an integration of seasonal processes which include, but are not limited to, net heat flux, stratification, nutrient depletion by primary producers, nutrient regeneration by benthic processes, and the release of POC and DOC from cell lysis and senescence. Altogether these processes lead to a smooth recurring pattern of increases and decreases in diversity not driven by temperature (kinetic energy hypothesis) per se or reflected in chl-a directly (resource hypothesis).

Furthermore, using the CLR transformation, we presented novel insights into the seasonal niche partitioning of conditionally rare phyla. In our study we provided evidence that five ecotypes of chemolithoautotrophic bacteria from the SUP05 lineage might be prime candidates for the dark C-fixation rates measured by Taguchi and Platt^60^. Insights into the seasonal changes of indicator species that perform distinct functions, such as ammonia oxidisers, sulfur reducers, bacterial primary producers such as Cyanobacteria, and those that form a close relationship with polysaccharide degradation, will allow us to further explore the correlative relationships of inferred functions. The inferred functions, we argue, have the potential to reveal new insights into how changes in bacterial diversity relate to energy production (in our study lipid biosynthesis pathways correlated with the productive spring bloom). Ultimately, we showed that metabarcoding data can be used to establish baselines of microbial diversity that can then be used to assess how natural and anthropogenic stressors impact the base of marine food webs.

## Materials and methods

### The Bedford Basin: sampling & oceanographic data

Bedford Basin is an estuary that forms the inner portion of Halifax Harbour, Nova Scotia, Canada. The Bedford area is known as Kwipek (Head of the Tide) to the Mi'kmaq First Nation people. Weekly water samples were collected at the Compass Buoy Station (HL0; 44.6936, −63.6403; Fig. 1a,b) in Bedford Basin, in association with the Bedford Basin Monitoring Program (https://www.bio.gc.ca/science/monitoring-monitorage/bbmp-pobb/bbmp-pobb-en.php). Using Niskin bottles, discrete samples were taken at depths of 1, 5, 10, and 60 m (Fig. 1b). The sampling depths in our study were chosen to align with the long-term oceanographic Bedford Basin Monitoring Program which has been collecting weekly physical, chemical and biological data since 1992. Seawater samples were maintained in the dark at cool temperatures and transported to nearby Dalhousie University for processing. Seawater (500 mL) from each depth was filtered onto 0.2 μm polycarbonate Isopore filters (Millipore, Ireland) with a mesh prefilter (160 μm in 2014–2015; 330 μm in 2016–2017). Filters were stored at −80 °C. CTD profiles performed by BIO and the CERC.OCEAN group at Dalhousie University recorded environmental measurements in conjunction with the collection of water samples for DNA.

### DNA extraction and sequencing

DNA was extracted from filtered cells using the DNeasy Plant Mini Kit (Qiagen, Germany) and the modified protocol described in Zorz et al.^66^. In brief, the lysis protocol was enhanced with a 5-min incubation with 50 µL lysozyme (5 mg/mL; Fisher BioReagents, UK) and a one hour incubation at 52 °C with Buffer AP1 and 45 µL Proteinase K (20 mg/mL; Fisher BioReagents, UK). At the conclusion of the manufacturer’s extraction protocol, DNA was eluted twice in 50 µL AE Buffer (Qiagen, Germany) and quantified with the NanoDrop 2000c (Thermo Scientific, US). The V4–V5 region of the *16S* ribosomal RNA was sequenced using the Illumina MiSeq (300 + 300 paired-end sequencing) at the Integrated Microbiome Resource (IMR; Dalhousie University, Halifax, NS); libraries were prepared by the IMR as in Comeau et al.^67^ using primers 515FB = GTGYCAGCMGCCGCGGTAA and 926R = CCGYCAATTYMTTTRAGTTT^68,69^.

### Amplicon sequence variants (ASVs)

Raw Illumina reads were processed using the Microbiome Helper amplicon pipeline^67^. In brief, primer sequences were removed using *cutadapt*^70^ and paired-end reads were stitched together using PEAR^71^. These sequences were imported into the QIIME2 2019.7 environment^72^ and low-quality reads were removed; the remaining reads were denoised into Amplicon Sequence Variants (ASVs) using deblur^73^ with a trim length of 350 bp. ASVs with sequence read frequencies less than [0.001 × mean sample depth] were attributed to sequencer bleed-through and removed. Taxonomy was assigned using the assignTaxonomy command in DADA2^74^ which uses a full-length *16S* Naïve Bayes-trained classifier based on the SILVA database (release 138^75,76^). Sequences which were assigned to chloroplasts and mitochondria were removed prior to the analyses.

### PICRUSt2

The software PICRUSt2 (version 2.4.1b^26,77^) was used with default settings to infer the functional potential of the microbial communities across the 4 year time series study. Chloroplasts and mitochondrial sequences were removed from the data set prior to analyses. The average Nearest Sequenced Taxon Index (NSTI) score, based on 793 samples (covering the 4 depths), was 0.155 ± 0.046 (± SD). Pathways with < 10 reads were removed from the data set. The final predicted metabolic pathway abundance data were centered log-ratio (CLR) transformed to plot seasonal trends.

### Statistical analyses

The Phyloseq (v.1.32.0; McMurdie and Holmes^78^), and microbiome (v. 1.7.21^79^) packages were used to analyse, visualize and plot the microbiome and physical and bio-chemical metadata using R (v4.0.2; Team^80^) in RStudio (v.1.3.1093). Plots were generated in R using ggplot2 or Ocean Data View version 4.6.5 unless stated otherwise^81,82^. Day lengths were calculated using the Geosphere package (v.1.5.10^83^) in R.

Statistical tests were conducted using the Vegan package version 2.5–6^45^. The Pearson’s correlation coefficient was used as a statistical measure to test the strength of the linear relationships between our paired data and to compare our findings to the results from Fuhrman et al. (2008). The plyr package (v.1.8.4) was used to calculate means and summarise the data^84^. The rarefaction curves and diversity measures were calculated using Phyloseq^78^ (v.1.32.0). Alpha diversity trends did not change when ASV tables were subsampled to different sampling depths of 5000, 8000, 10,000 and 20,000 sequences per sample (Supplementary Figs. 3 and 4). Multiple regression models were conducted using the Vegan package^45^ (v.2.5–6). The relaimpo package with the calc.relimp function was used to calculate the relative importance of each parameter to the model (v. 2.2.5; Groemping and Matthias^85^). Analysis of similarities (ANOSIM) were used to test whether we could identify statistical differences between the four depths, the years and different seasons using the PRIMER-e version 7.0.17^86^. Prior to ANOSIM analyses, the community matrices were CLR transformed and Aitchison distances were generated using the Vegan package in R^45^.

Redundancy analyses (RDAs; using CLR transformed ASV tables) were used to characterize the specific abiotic variables in the water column which exerted the largest influence on the prokaryotic community compositions. Environmental parameters were standardized with the ‘standardize’ function (variables were scaled to zero mean and unit variance) using decostand from the Vegan Package^45^ and significant (*p* < 0.05) environmental parameters were derived using the ‘envfit’ function in Vegan and overlaid as vectors to identify multiple explanatory variables between the estuarine zones^45^. The ‘indicspecies’ package (ver. 1.7.8;^52^) with the ‘multipatt’ function and 9999 permutations was used to identify prokaryotic indicators within each month. ASV tables were agglomerated at a Phylum level (using Phyloseq (v.1.32.0) and the abundance tables were then CLR transformed. Pearson’s phi coefficients of association^87^ were calculated to determine significant indicator pathways across the different months. The phi coefficient was corrected using the function = ”r.g.” to accommodate for the fact that some months had more sampling points than others^88^. To account for compositionality of sequencing data^49^, the trends in seasonal changes of the prokaryotic indicators were plotted using CLR values rather than relative abundances. The heatmap was produced using the *ampvis2* R package.

## Supplementary Information

Supplementary Information 1.

Supplementary Information 2.

## Data Availability

All physical and biogeochemical water column data are available through the Bedford Basin Monitoring Program (https://www.bio.gc.ca/science/monitoring-monitorage/bbmp-pobb/bbmp-pobb-en.php ). Sequence data are archived at the NCBI under BioProject ID PRJNA785606. Sequences for the five SUP05 ecotypes are available under GenBank accession numbers MZ890602, MZ890603, MZ890604, MZ890605 and MZ890606. Scripts to reproduce the figures along with the ASV table, metadata and taxonomy are available at https://github.com/EricRaes/Bedford_Basin_Time_Series.
