# Supplementary material for: Seasonal bacterial niche structures and chemolithoautotrophic ecotypes in a North Atlantic fjord
Source: Sci Rep. 2022 Sep 12;12:15335. doi: 10.1038/s41598-022-19165-w (PMC9468339; doi:10.1038/s41598-022-19165-w)
Supplement: Supplementary file 1 — Supplementary Information 1. [file 41598_2022_19165_MOESM1_ESM.docx]

**Supplementary Material**

**Seasonal bacterial niche structures and chemolithoautotrophic ecotypes in a North Atlantic fjord**

Eric J. Raes^1,2^, Jennifer Tolman^1^, Dhwani Desai^1,3^, Jenni-Marie Ratten^1^, Jackie Zorz^4^, Brent M. Robicheau^1^, Diana Haider^1,3^, Julie LaRoche^1*^

1. Dept. of Biology, Dalhousie University, Halifax, NS, B3H 4R2, Canada
2. Flourishing Oceans, Minderoo Foundation, Broadway, 6009, WA, Australia
3. Dept. of Pharmacology, Dalhousie University, Halifax, NS, B3H 4R2, Canada
4. Dept. of Geoscience, University of Calgary, Calgary, AB, T2N 1N4, Canada
5. Faculty of Computer Science, Dalhousie University, Halifax, NS, B3H 4R2, Canada

*** Corresponding authors:** [Julie.LaRoche@dal.ca](mailto:Julie.LaRoche@dal.ca)

**Supplementary Figures:**


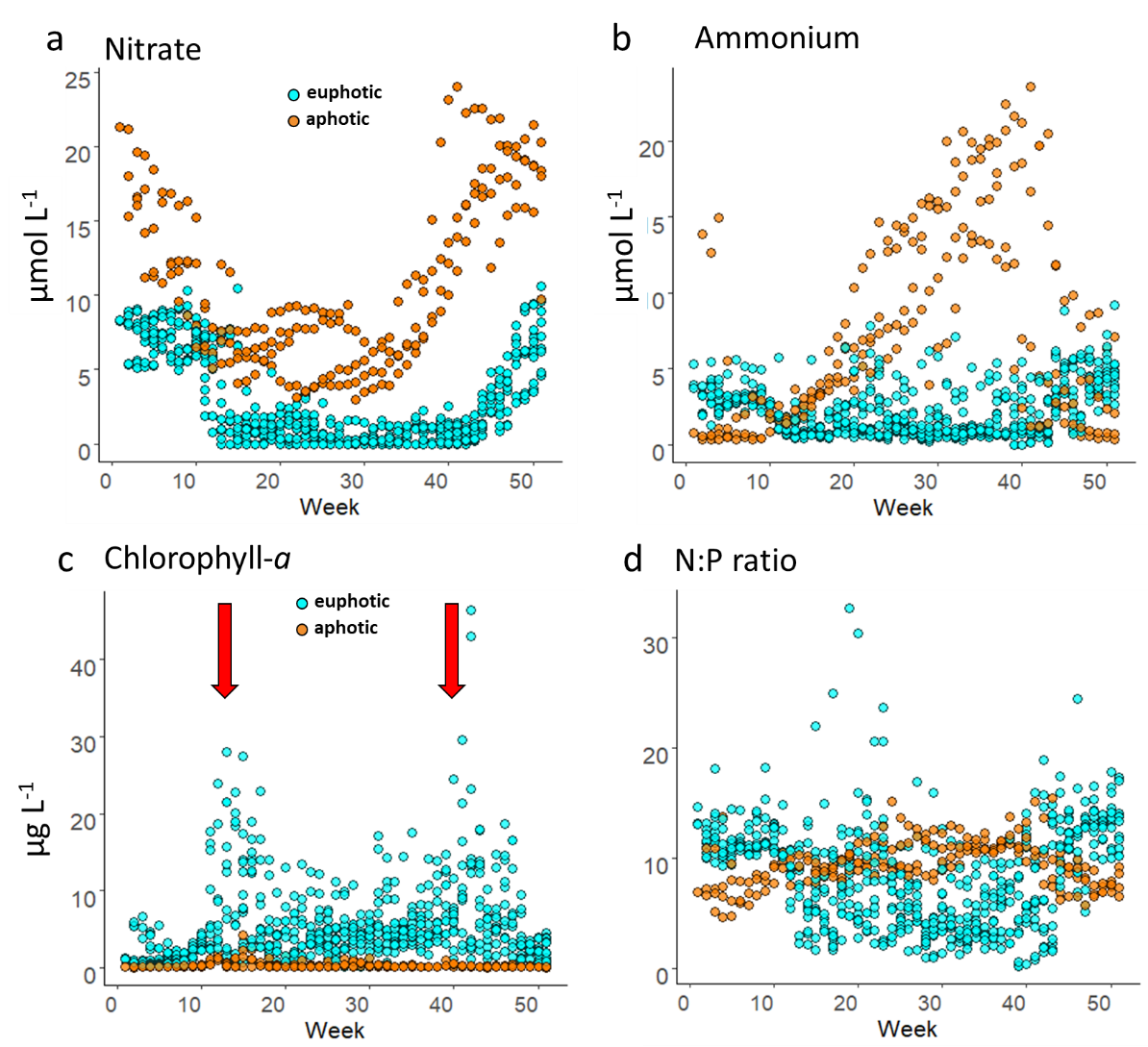


**Supplementary Figure 1:** Seasonal trend in nitrate (**a**), ammonium (**b**), chlorophyll-*a* (**c**), and N:P ratios (N is the sum of nitrate + ammonium and nitrite; **d**) in the Bedford Basin from 2014-2017. Sampling depths are colour coded for the euphotic (1, 5 and 10m; cyan circles) and aphotic zones (60m; orange circles Red arrows (**c**) denote the spring and autumn blooms.


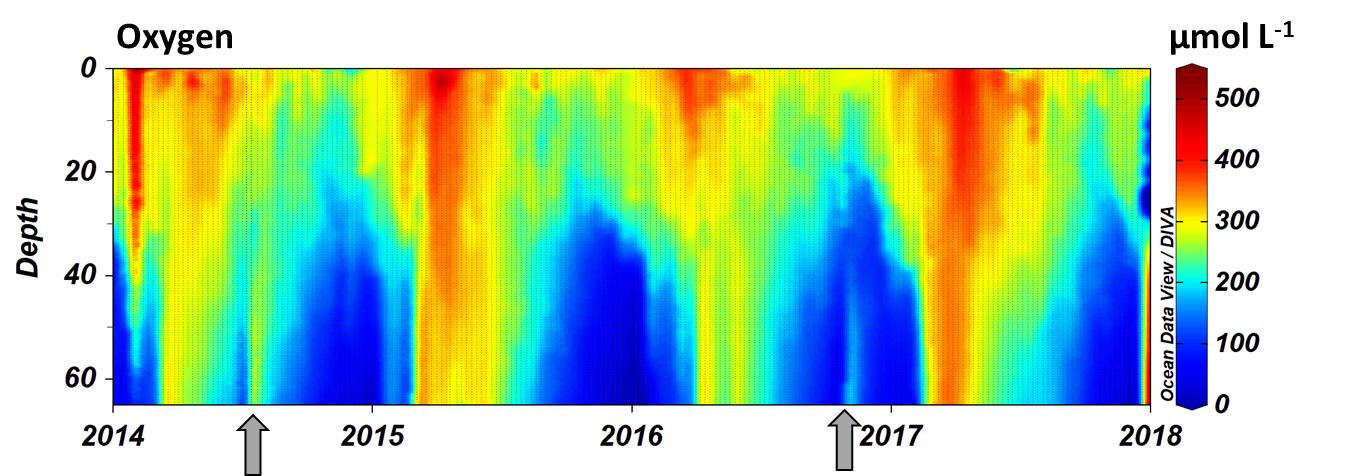


**Supplementary Figure 2:** Seasonal dissolved oxygen concentrations (in µmol L^-1^), with suboxic concentrations in the deeper water in the colder winter months. Intrusion events in July 2014 and November 2017 are marked by grey arrows.


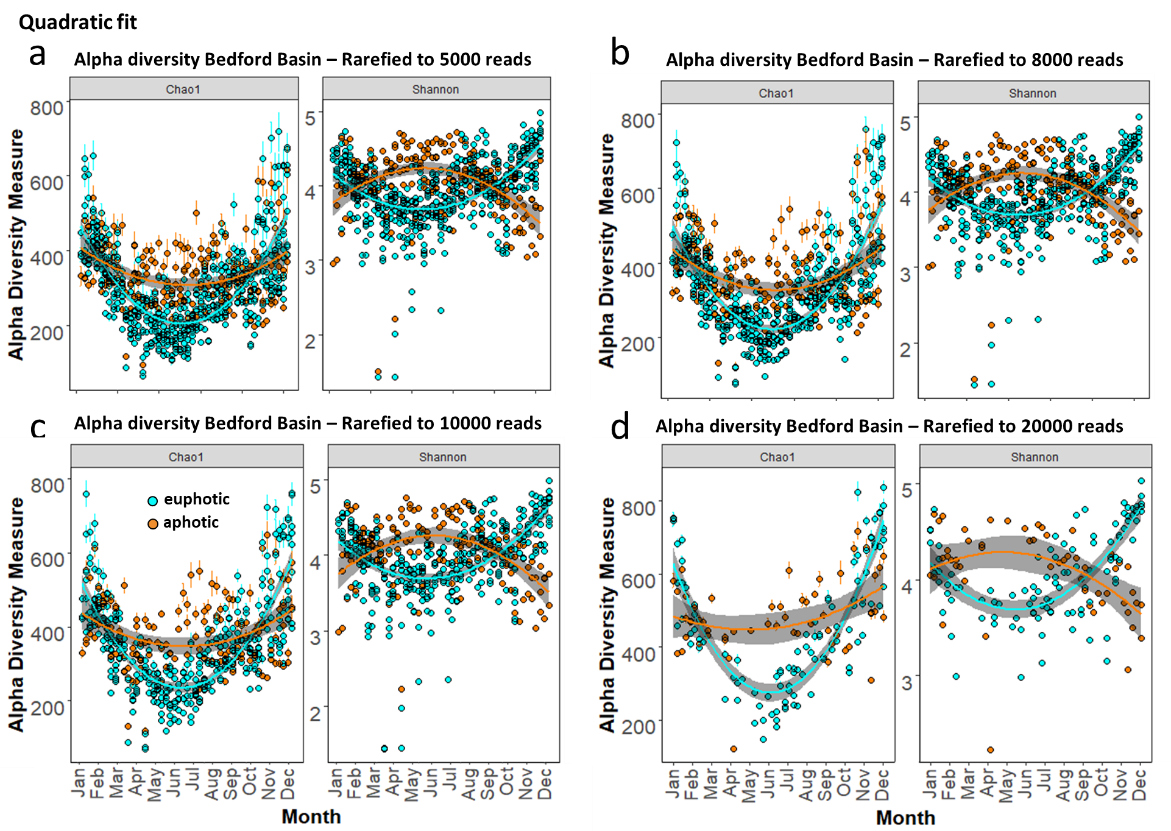


**Supplementary Figure 3:** Alpha diversity, both Chao1 and Shannon, after different rarefaction depths. **a**) ASV table rarefied to 5000 reads, **b**) rarefied to 8000 reads, **c**) rarefied to 10000 reads and **d**) rarefied to 20000 reads. The seasonal trends remain regardless of rarefaction depth. Quadratic fits are added for the photic and aphotic zones.


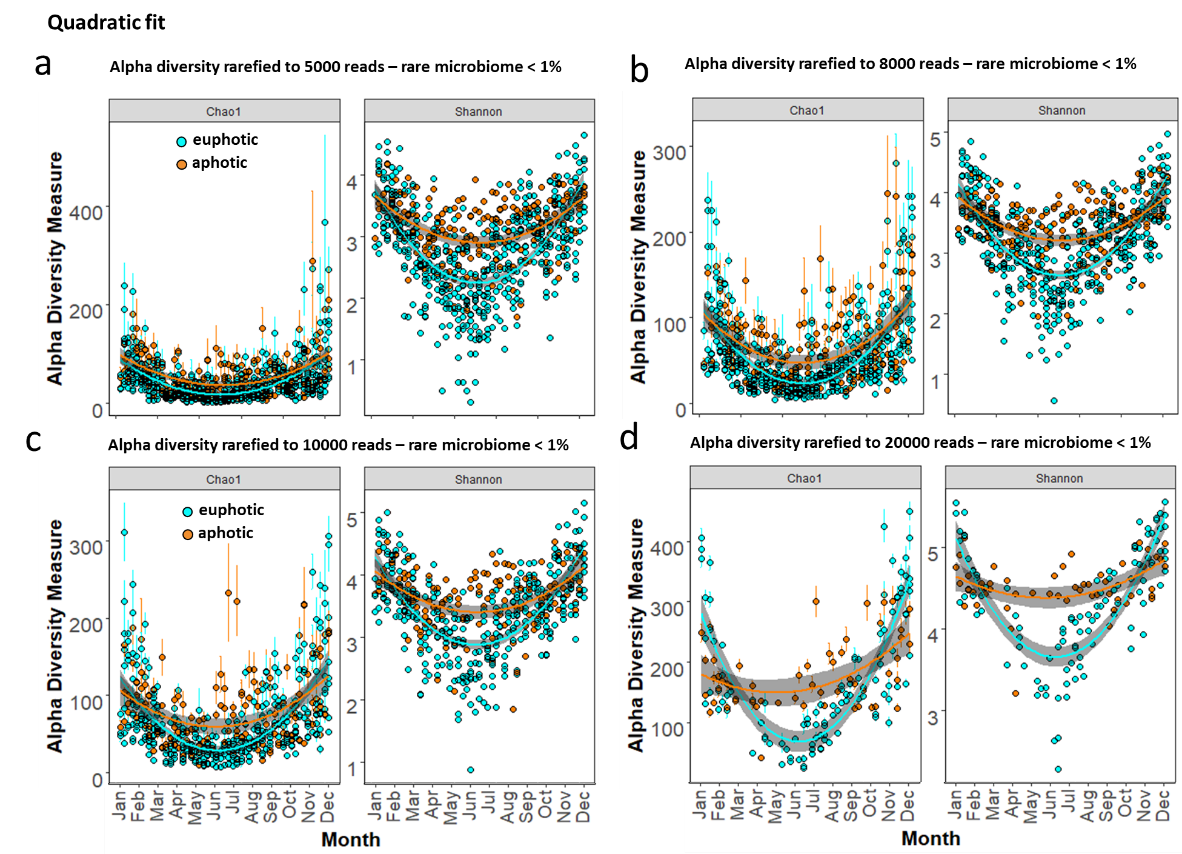


**Supplementary Figure 4:** Alpha diversity for the rare microbiome (<1% of ASV reads), both Chao1 and Shannon, after different rarefaction depths. **a**) ASV table rarefied to 5000 reads, **b**) rarefied to 8000 reads, **c**) rarefied to 10000 reads and **d**) rarefied to 20000 reads. The seasonal trends remain regardless of rarefaction depths. Quadratic fits are added for the photic and aphotic zones.

**Extra note:**


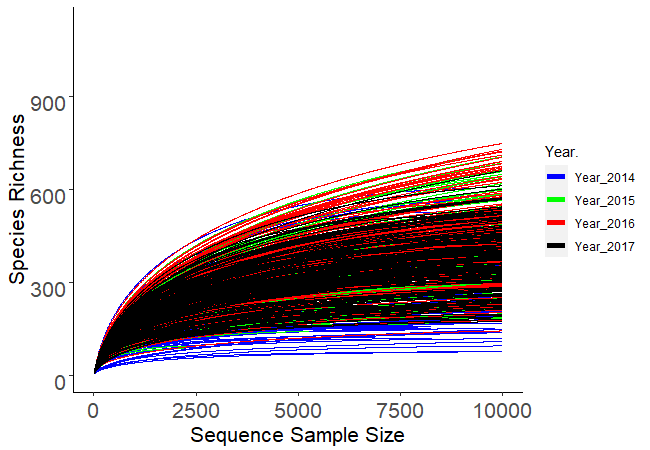
Rarefaction curves plateaued for most samples at 5,000 reads (please see the figure on the right).

A slight increase in the rarefaction depth could report ‘*truer’* values but our findings and overall results (i.e., strong seasonal changes) would not change, hence we rather refer the reader to the supplementary figure above where we display the seasonal trends across different rarefaction depths.

Additionally, 196 samples are removed due to insufficient reads when we rarefy to 10,000 reads, whereas only 64 samples are removed when we rarefy to 5,000 reads. We prefer to keep a higher number of samples and report the seasonal trends at different rarefaction depths in the Supplementary material.


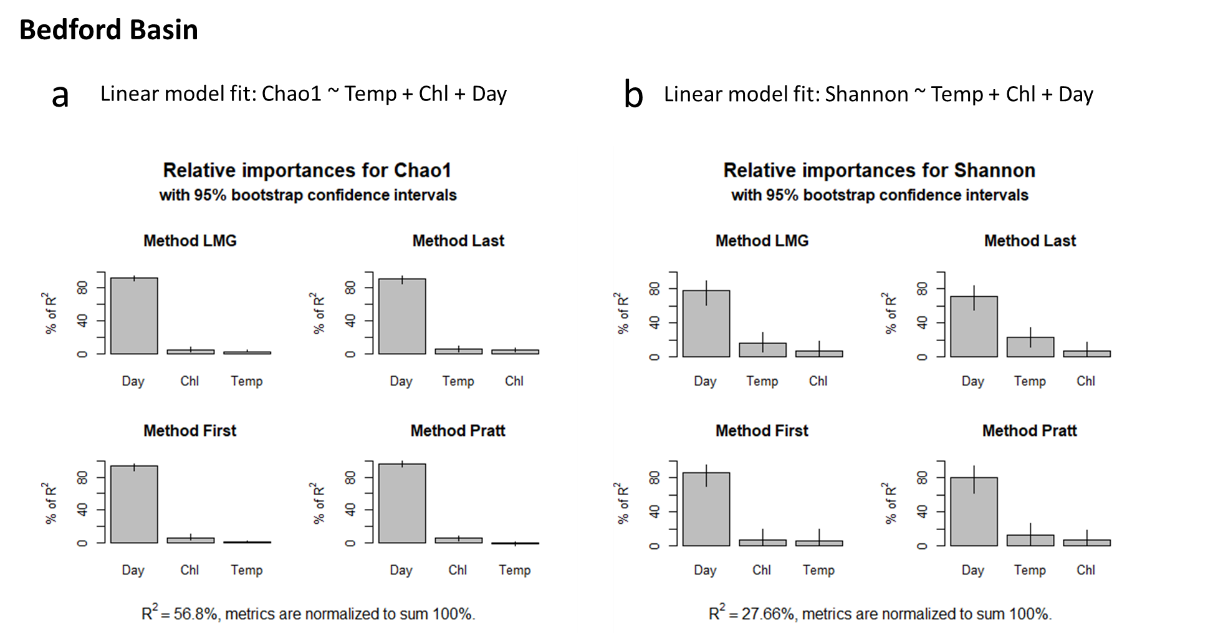


**Supplementary Figure 5:** Multiple linear regression models (with temperature + chl-a + day length fitted last) for the prokaryotic community in the euphotic zone. Linear regression models were conducted using the Vegan package (v.2.5-6; Oksanen *et al.*, 2007) and the relaimpo package (v. 2.2.5; Groemping and Matthias (2021)) with the calc.relimp function was used to calculate the relative importance of each parameter to the model.


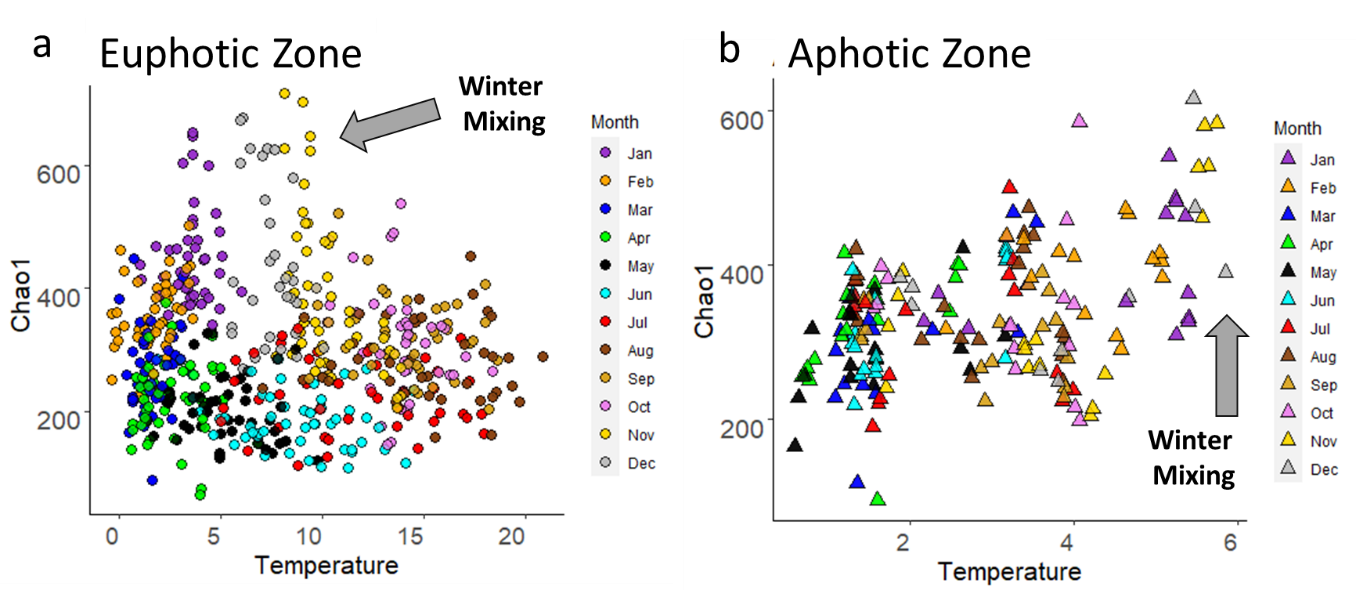


**Supplementary Figure 6:** Alpha diversity trends in the Bedford Basin from 2014-2017. Species richness (Chao1 diversity) correlated with temperature in the euphotic and aphotic zone (**a** and **b;** rarefied to 5000 reads). Grey arrows denote winter mixing and highest diversity in the winter.


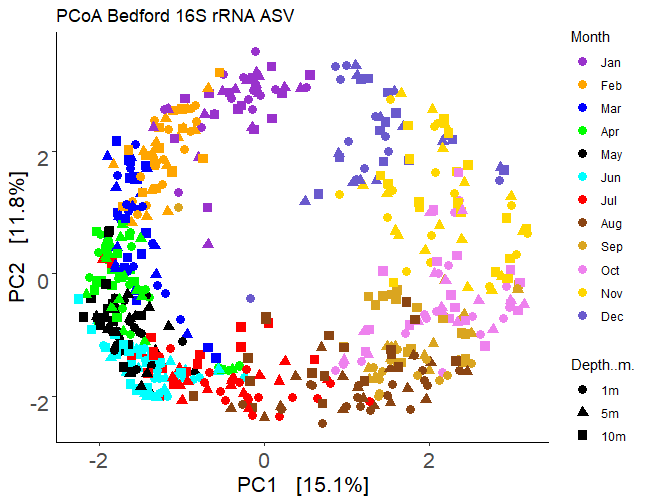


**Supplementary Figure 7:** Unconstrained PCA with CLR transformed ASVs and Aitchison (Euclidian) distance matrix in the euphotic zone. Symbols denote different depths, and colour different months.


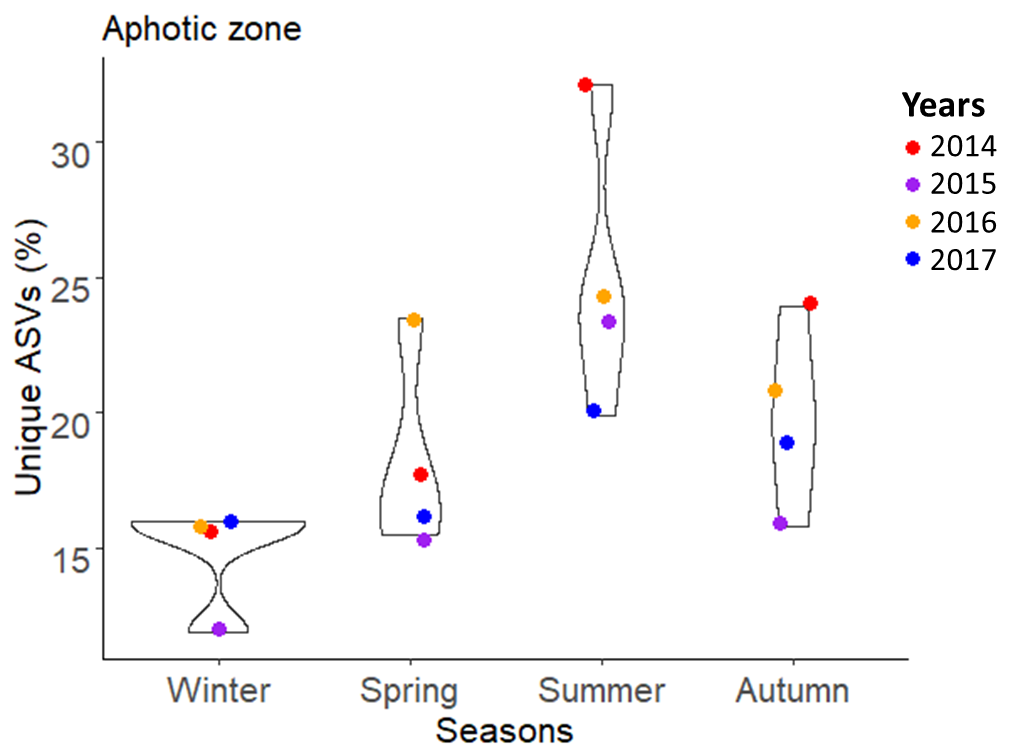


**Supplementary Figure 8:**  Unique ASVs in the aphotic zone (expressed as a percentage). Note the doubling of unique ASVs in the aphotic zone in summer due to stratification of the water column. Unique ASVs were derived using the ggven package v.0.1.8 in R (Yan, L. ggvenn: draw Venn diagram by ‘ggplot2’; https://CRAN.R-project.org/package=ggvenn (2020)).


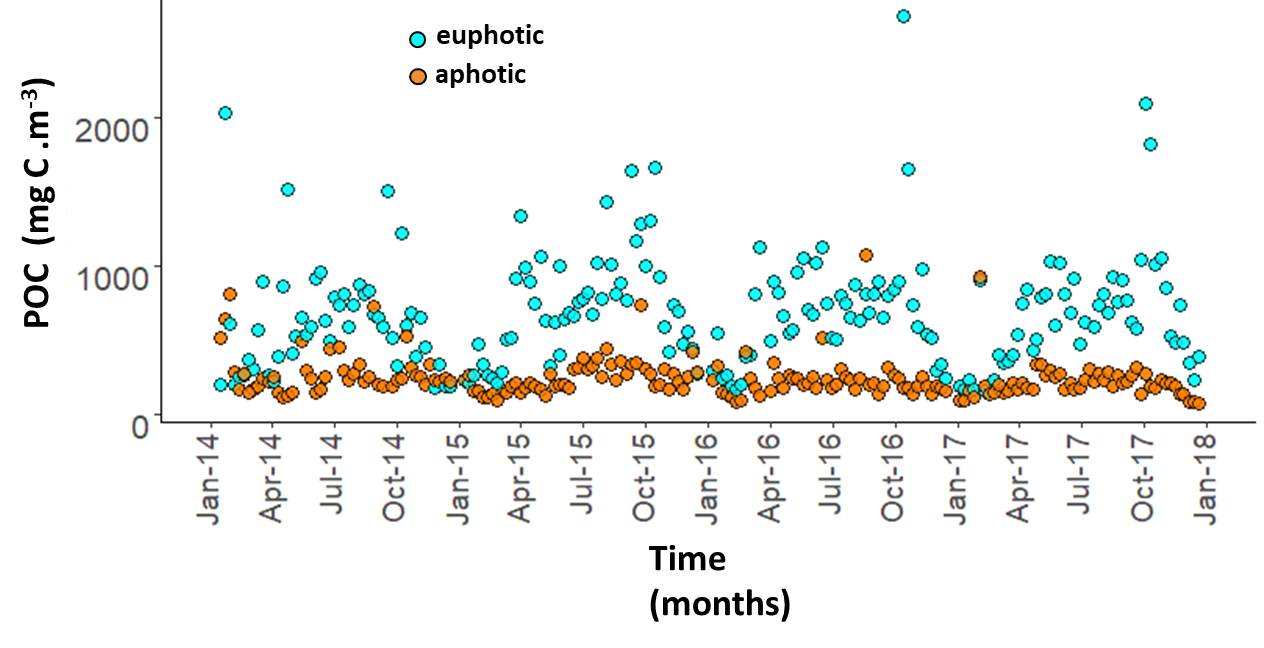


**Supplementary Figure 9:** Seasonal changes in particulate organic matter (POC). Note no samples were collected at 1m depth.


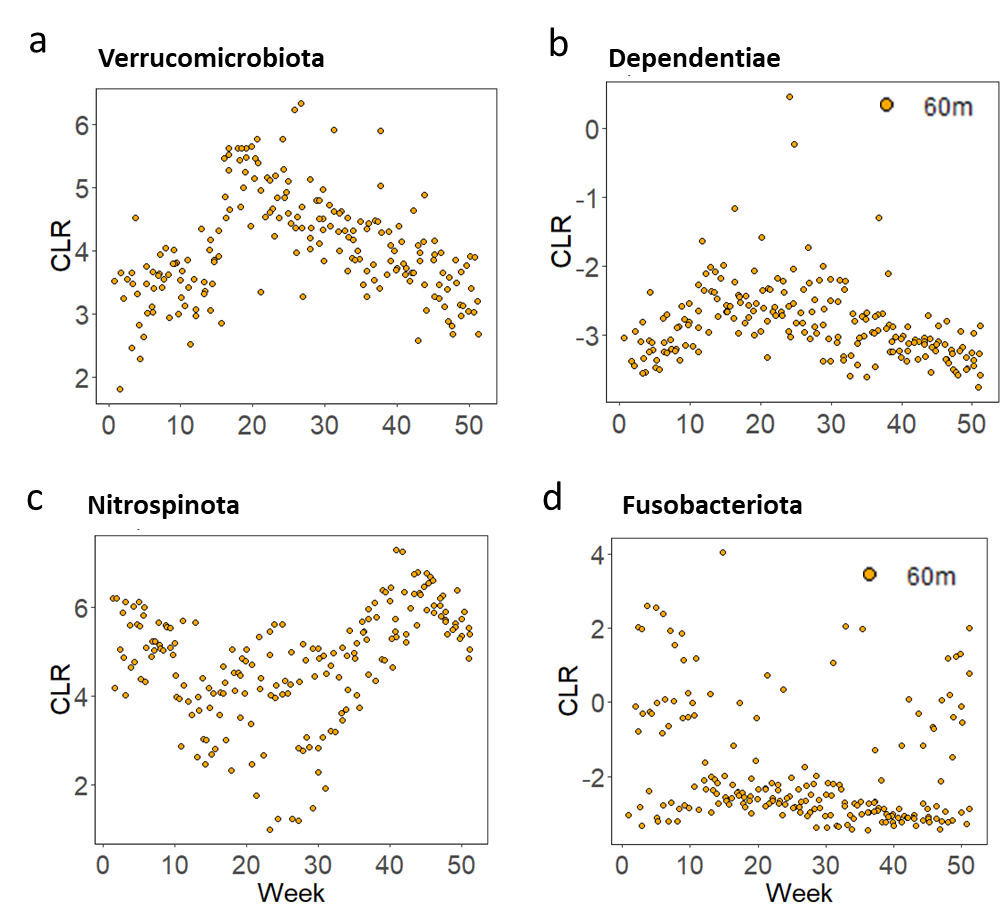


**Supplementary Figure 10:** Seasonal changes in bacterial phyla in the aphotic zone (60 m) in Bedford Basin. Significance patterns and temporal niches were assessed with a permutation test (“multipatt” function; see Methods and supplementary Table 10). Due to the compositional nature of the sequence data the changes are visualized with CLR values rather than relative abundances.


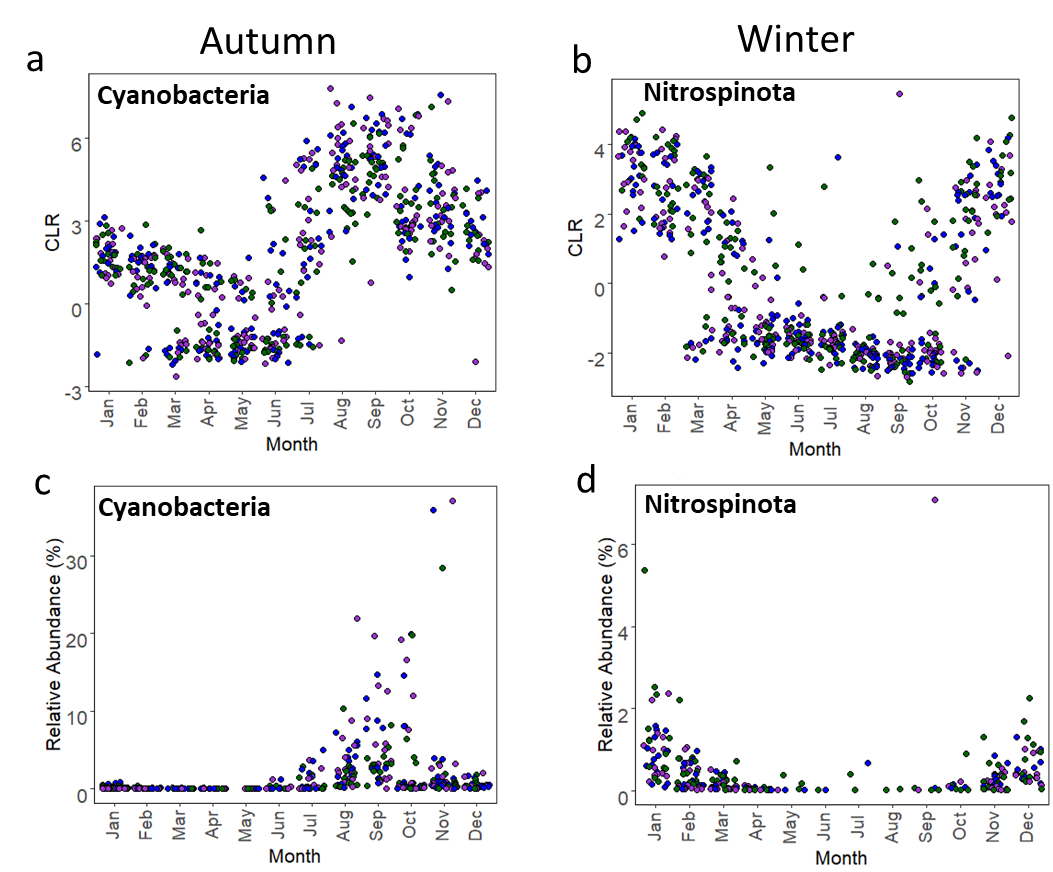


**Supplementary Figure 11:** Seasonal trends in the euphotic zone for Cyanobacteria and Nitrospinota shown as CLR transformed data (**a** and **b**), and relative abundances (**c** and **d**) in the Bedford basin across a 4-year time series data set. Rarefaction depth was 5000 reads.


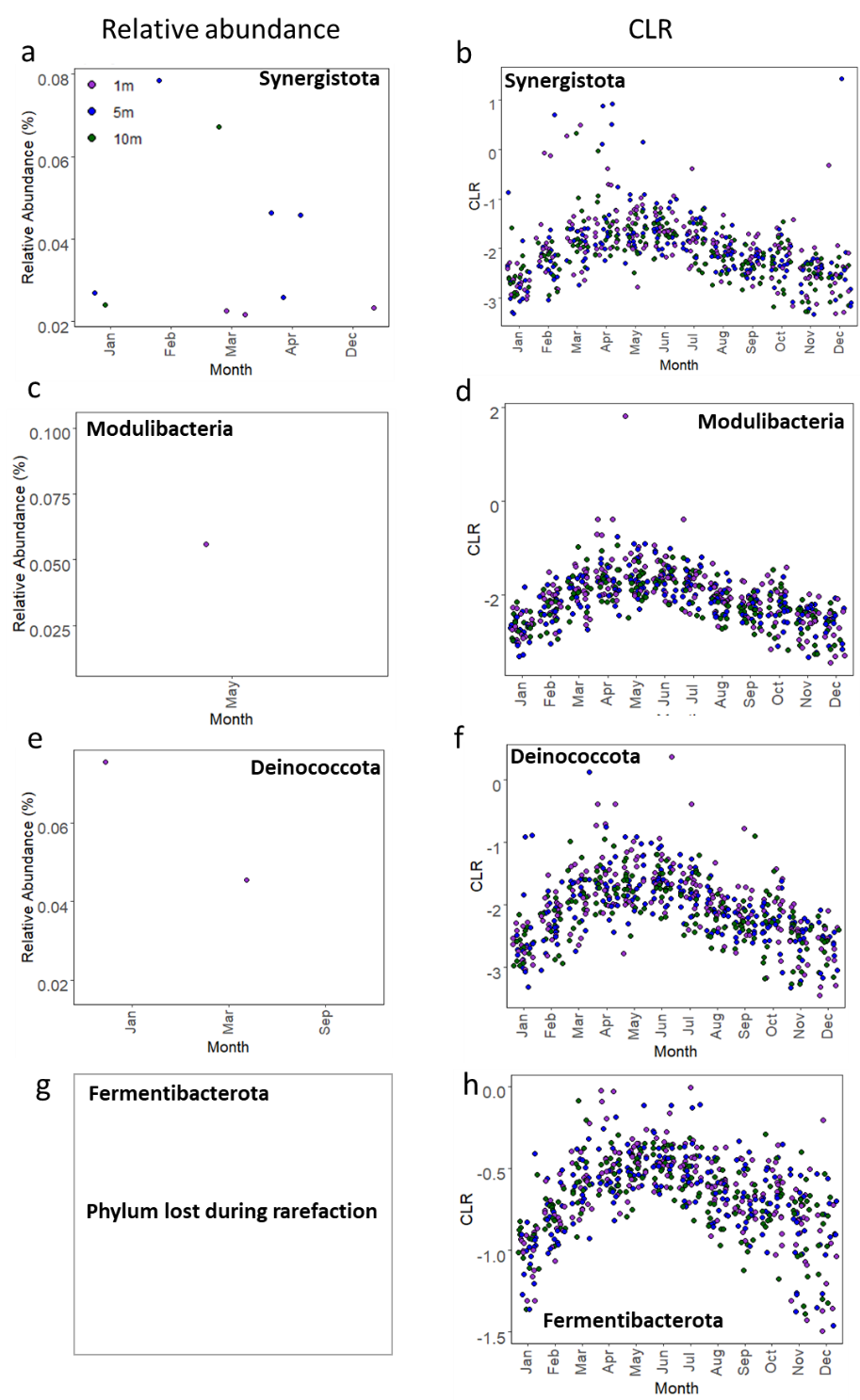


**Supplementary Figure 12:** Seasonal trends disappear when the ASV table was rarefied to a depth of 5000 reads and expressed as relative abundances. The rare phyla (which contributed < 0.1% of the relative bacterial abundance) either did not show any seasonal trends or were removed during rarefaction. Relative abundances and CLR trends for **a-b**) Syngergistota, **c-d**) Modulibacteria, **e-f**) Deinococcota and **g-h**) Fermentibacterota.


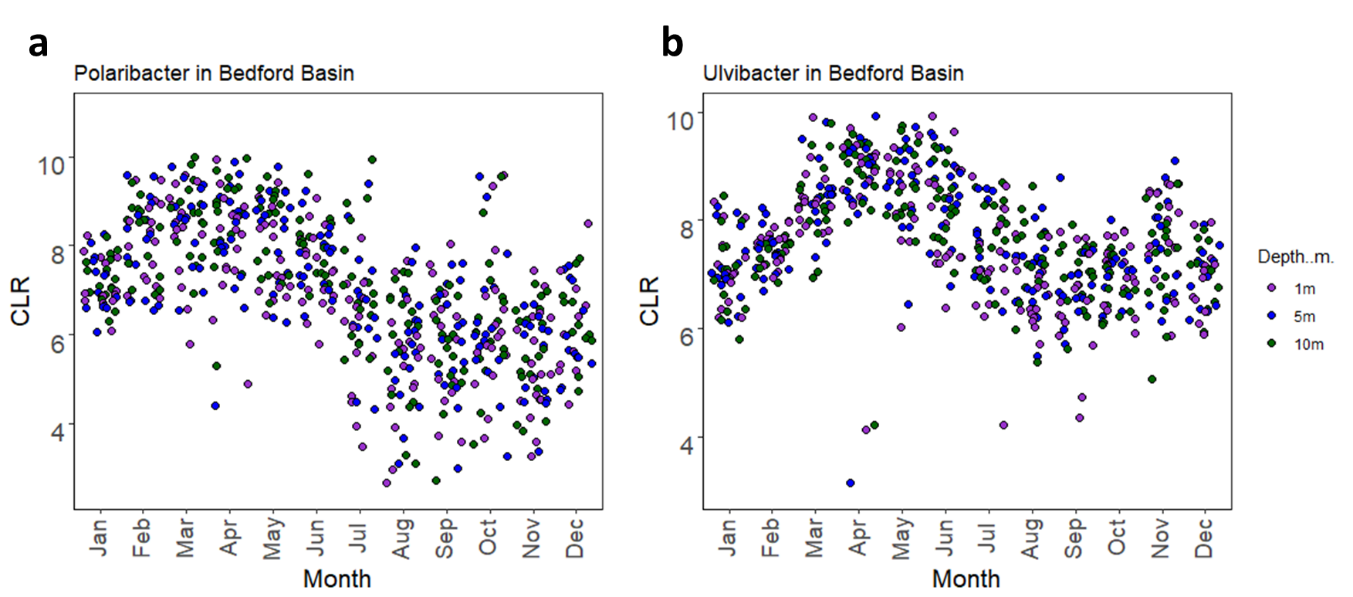


**Supplementary Figure 13:** Seasonal trends in the euphotic zone for Pelagibacter and Ulvibacter are associated with the spring bloom in the Bedford basin across a 4-year time series data set. Data were CLR transformed.

**Supplementary Tables**

**Supplementary Table 1:**  Yearly salinity value (PSU).

| Salinity |  |  |  |  |
| --- | --- | --- | --- | --- |
| Year | **mean** | **sd** | **max** | **min** |
| 2014 | 30.04665 | 0.970396 | 31.2700 | 25.5500 |
| 2015 | 30.01835 | 0.917605 | 31.3576 | 26.4968 |
| 2016 | 30.18413 | 0.738362 | 31.3098 | 27.5186 |
| 2017 | 30.13276 | 0.908307 | 31.5833 | 25.4061 |
|  |  |  |  |  |
| Salinity |  |  |  |  |
| group1 | **group2** | **p** | **signif** | **method** |
| 2016 | 2017 | 0.863 | ns | Wilcoxon |
| 2016 | 2014 | 0.377 | ns | Wilcoxon |
| 2016 | 2015 | 0.215 | ns | Wilcoxon |
| 2017 | 2014 | 0.491 | ns | Wilcoxon |
| 2017 | 2015 | 0.131 | ns | Wilcoxon |
| 2014 | 2015 | 0.645 | ns | Wilcoxon |

ns = not significant

**Supplementary Table 2:**  Quadratic fits to test the significance of the trends observed at each rarefaction depth in the euphotic and aphotic zone.

| **Quadratic fits** |  | **euphotic zone** | | | |
| --- | --- | --- | --- | --- | --- |
| Rarefied depth | Chao1 | Multiple R-squared | Adjusted R-squared | F-statistic | p-value |
| 5000 |  | 0.542 | 0.54 | 306.7 on 2 and 518 DF | < 2.2e-16 |
| 8000 |  | 0.579 | 0.577 | 300.1 on 2 and 436 DF | < 2.2e-16 |
| 10000 |  | 0.599 | 0.59 | 268.7 on 2 and 370 DF | < 2.2e-16 |
| 20000 |  | 0.787 | 0.783 | 188.9 on 2 and 102 DF | < 2.2e-16 |
| Rarefied depth | Shannon | Multiple R-squared | Adjusted R-squared | F-statistic | p-value |
| 5000 |  | 0.282 | 0.279 | 101.9 on 2 and 518 DF | < 2.2e-16 |
| 8000 |  | 0.295 | 0.291 | 91.1 on 2 and 436 DF | < 2.2e-16 |
| 10000 |  | 0.312 | 0.308 | 83.81 on 2 and 370 DF | < 2.2e-16 |
| 20000 |  | 0.534 | 0.525 | 58.48 on 2 and 102 DF | < 2.2e-16 |
|  |  |  |  |  |  |
| **Quadratic fits** |  | **aphotic zone** | | | |
| Rarefied depth | Chao1 | Multiple R-squared | Adjusted R-squared | F-statistic | p-value |
| 5000 |  | 0.107 | 0.097 | 11.4 on 2 and 190 DF | 2.11E-05 |
| 8000 |  | 0.122 | 0.112 | 11.9 on 2 and 171 DF | 1.43E-05 |
| 10000 |  | 0.091 | 0.078 | 7.34 on 2 and 147 DF | 8.87E-04 |
| 20000 |  | 0.13 | 0.097 | 3.977 on 2 and 53 DF | 0.02459 |
| Rarefied depth | Shannon | Multiple R-squared | Adjusted R-squared | F-statistic | p-value |
| 5000 |  | 0.195 | 0.184 | 22.64 on 2 and 190 DF | 1.51E-09 |
| 8000 |  | 0.195 | 0.186 | 20.71 on 2 and 171 DF | 8.81E-09 |
| 10000 |  | 0.176 | 0.165 | 15.71 on 2 and 147 DF | 6.58E-07 |
| 20000 |  | 0.192 | 0.162 | 6.3 on 2 and 53 DF | 0.00351 |

**Supplementary Table 3:**  Seasonal alpha diversity comparisons in the euphotic zone.

| Diversity | group1 | group2 | p | p-adjusted | significance | method |
| --- | --- | --- | --- | --- | --- | --- |
| Chao1 | Winter | Spring | 6.64E-39 | 4.00E-38 | **** | Wilcoxon |
| Chao1 | Winter | Summer | 7.47E-34 | 4.50E-33 | **** | Wilcoxon |
| Chao1 | Winter | Autumn | 1.03E-10 | 6.20E-10 | **** | Wilcoxon |
| Chao1 | Spring | Summer | 0.559572 | 1 | **ns** | Wilcoxon |
| Chao1 | Spring | Autumn | 2.84E-18 | 1.70E-17 | **** | Wilcoxon |
| Chao1 | Summer | Autumn | 7.73E-15 | 4.60E-14 | **** | Wilcoxon |
|  |  |  |  |  |  |  |
| Diversity | **group1** | **group2** | **p** | **p-adjusted** | **significance** | **method** |
| Shannon | Winter | Spring | 1.16E-13 | 7.00E-13 | **** | Wilcoxon |
| Shannon | Winter | Summer | 4.17E-06 | 2.50E-05 | **** | Wilcoxon |
| Shannon | Winter | Autumn | 0.010687 | 0.064 | **ns** | Wilcoxon |
| Shannon | Spring | Summer | 5.68E-05 | 0.00034 | **** | Wilcoxon |
| Shannon | Spring | Autumn | 4.16E-10 | 2.50E-09 | **** | Wilcoxon |
| Shannon | Summer | Autumn | 0.007546 | 0.045 | ** | Wilcoxon |

**ns** = not significant ** p <0.01; *** p < 0.001; **** p<0.0001

**Supplementary Table 4:**  Seasonal alpha diversity comparisons in the aphotic zone.

| **Diversity** | **group1** | **group2** | **p** | **p-adjusted** | **p-value** | **significance** | **method** |
| --- | --- | --- | --- | --- | --- | --- | --- |
| **Chao1** | Winter | Spring | 9.26E-06 | 5.60E-05 | 9.30E-06 | **** | Wilcoxon |
| **Chao1** | Winter | Summer | 0.001164 | 0.007 | 0.00116 | ** | Wilcoxon |
| **Chao1** | Winter | Autumn | 0.000333 | 0.002 | 0.00033 | *** | Wilcoxon |
| **Chao1** | Spring | Summer | 0.195674 | 1 | 0.19567 | **ns** | Wilcoxon |
| **Chao1** | Spring | Autumn | 0.516889 | 1 | 0.51689 | **ns** | Wilcoxon |
| **Chao1** | Summer | Autumn | 0.629631 | 1 | 0.62963 | **ns** | Wilcoxon |
|  |  |  |  |  |  |  |  |
| **Diversity** | **group1** | **group2** | **p** | **p-adjusted** | **p-value** | **significance** | **method** |
| **Shannon** | Winter | Spring | 0.001793 | 0.011 | 0.00179 | ** | Wilcoxon |
| **Shannon** | Winter | Summer | 0.000193 | 0.0012 | 0.00019 | *** | Wilcoxon |
| **Shannon** | Winter | Autumn | 0.399748 | 1 | 0.39975 | **ns** | Wilcoxon |
| **Shannon** | Spring | Summer | 0.494764 | 1 | 0.49476 | **ns** | Wilcoxon |
| **Shannon** | Spring | Autumn | 4.53E-06 | 2.70E-05 | 4.50E-06 | **** | Wilcoxon |
| **Shannon** | Summer | Autumn | 3.28E-08 | 2E-07 | 3.3E-08 | **** | Wilcoxon |

**ns** = not significant ** p <0.01; *** p < 0.001; **** p<0.0001

| **Quadratic fits** | | **euphotic zone** | | | |
| --- | --- | --- | --- | --- | --- |
| Rarefied depth | Chao1 | Multiple R-squared | Adjusted R-squared | F-statistic | p-value |
| 5000 |  | 0.542 | 0.54 | 306.7 on 2 and 518 DF | < 2.2e-16 |
| 8000 |  | 0.579 | 0.577 | 300.1 on 2 and 436 DF | < 2.2e-16 |
| 10000 |  | 0.599 | 0.59 | 268.7 on 2 and 370 DF | < 2.2e-16 |
| 20000 |  | 0.787 | 0.783 | 188.9 on 2 and 102 DF | < 2.2e-16 |
| Rarefied depth | Shannon | Multiple R-squared | Adjusted R-squared | F-statistic | p-value |
| 5000 |  | 0.282 | 0.279 | 101.9 on 2 and 518 DF | < 2.2e-16 |
| 8000 |  | 0.295 | 0.291 | 91.1 on 2 and 436 DF | < 2.2e-16 |
| 10000 |  | 0.312 | 0.308 | 83.81 on 2 and 370 DF | < 2.2e-16 |
| 20000 |  | 0.534 | 0.525 | 58.48 on 2 and 102 DF | < 2.2e-16 |
|  |  |  |  |  |  |
| **Quadratic fits** | | **aphotic zone** | | | |
| Rarefied depth | Chao1 | Multiple R-squared | Adjusted R-squared | F-statistic | p-value |
| 5000 |  | 0.107 | 0.097 | 11.4 on 2 and 190 DF | 2.11E-05 |
| 8000 |  | 0.122 | 0.112 | 11.9 on 2 and 171 DF | 1.43E-05 |
| 10000 |  | 0.091 | 0.078 | 7.34 on 2 and 147 DF | 8.87E-04 |
| 20000 |  | 0.13 | 0.097 | 3.977 on 2 and 53 DF | 0.02459 |
| Rarefied depth | Shannon | Multiple R-squared | Adjusted R-squared | F-statistic | p-value |
| 5000 |  | 0.195 | 0.184 | 22.64 on 2 and 190 DF | 1.51E-09 |
| 8000 |  | 0.195 | 0.186 | 20.71 on 2 and 171 DF | 8.81E-09 |
| 10000 |  | 0.176 | 0.165 | 15.71 on 2 and 147 DF | 6.58E-07 |
| 20000 |  | 0.192 | 0.162 | 6.3 on 2 and 53 DF | 0.00351 |

**Supplementary Table 5:** Correlating environmental parameters (derived from envfit; Vegan package; (Oksanen et al., 2007)) with ASV table.

| **Parameter** | **PC1** | **PC2** | **r2** | **p-value** |
| --- | --- | --- | --- | --- |
| **Day Length** | -0.45788 | -0.88901 | 0.9083 | 0.001 |
| **Temp** | 0.78568 | -0.61864 | 0.8313 | 0.001 |
| **NO3** | -0.13139 | 0.99133 | 0.7095 | 0.001 |
| **PO4** | -0.06238 | 0.99805 | 0.6733 | 0.001 |
| **Si** | -0.20484 | 0.97879 | 0.6204 | 0.001 |
| **DO** | -0.99998 | 0.00575 | 0.5119 | 0.001 |
| **Chl-a** | 0.5048 | -0.86324 | 0.0722 | 0.001 |
| **Sal** | 0.75152 | -0.65971 | 0.0382 | 0.001 |

Permutations = 999

**Supplementary Table 6:** ANOSIM results for depth group comparisons, nested within months and shown for all 4 years. Global tests and pairwise group tests respectively. ASV data were CLR transformed with an Aitchison distance matrix.

|  | **2014** | **2015** | **2016** | **2017** |
| --- | --- | --- | --- | --- |
| **Global test (R)** | 0.297 | 0.235 | 0.333 | 0.299 |
| **Significance** | **0.001** | **0.001** | **0.001** | **0.001** |
| **Permutations** | 999 | 999 | 999 | 999 |

| **Year** | **Depth Group** | **R-value** | **Significance** | **Permutations** |
| --- | --- | --- | --- | --- |
| **2014** | **1m-5m** | -0.048 | 0.838 | 999 |
|  | **1m-10m** | -0.019 | 0.537 | 999 |
|  | **1m-60m** | 0.676 | **0.001** | 999 |
|  | **5m-10m** | -0.034 | 0.66 | 999 |
|  | **5m-60m** | 0.632 | **0.001** | 999 |
|  | **10m-60m** | 0.535 | **0.001** | 999 |
|  |  |  |  |  |
| **2015** | **1m-5m** | -0.055 | 0.878 | 999 |
|  | **1m-10m** | 0.009 | 0.337 | 999 |
|  | **1m-60m** | 0.529 | **0.001** | 999 |
|  | **5m-10m** | -0.029 | 0.594 | 999 |
|  | **5m-60m** | 0.499 | **0.001** | 999 |
|  | **10m-60m** | 0.398 | **0.001** | 999 |
|  |  |  |  |  |
| **2016** | **1m-5m** | -0.023 | 0.553 | 999 |
|  | **1m-10m** | 0.008 | 0.376 | 999 |
|  | **1m-60m** | 0.708 | **0.001** | 999 |
|  | **5m-10m** | -0.018 | 0.526 | 999 |
|  | **5m-60m** | 0.692 | **0.001** | 999 |
|  | **10m-60m** | 0.6 | **0.001** | 999 |
|  |  |  |  |  |
| **2017** | **1m-5m** | -0.057 | 0.92 | 999 |
|  | **1m-10m** | 0.544 | **0.001** | 999 |
|  | **1m-60m** | -0.037 | 0.676 | 999 |
|  | **5m-10m** | -0.05 | 0.831 | 999 |
|  | **5m-60m** | 0.532 | **0.001** | 999 |
|  | **10m-60m** | 0.43 | **0.001** | 999 |

Significant results are marked in red.

**Supplementary Table 7:** Global ANOSIM results testing monthly differences in the bacterial communities in the euphotic and aphotic zones. ASV data were CLR transformed with an Aitchison distance matrix.

|  | **Global test (R)** | |  |  |
| --- | --- | --- | --- | --- |
| **Years** | **Euphotic** | **Aphotic** | **Significance** | **Permutations** |
| **2014** | 0.734 | 0.731 | 0.001 | 999 |
| **2015** | 0.803 | 0.83 | 0.001 | 999 |
| **2016** | 0.806 | 0.719 | 0.001 | 999 |
| **2017** | 0.842 | 0.77 | 0.001 | 999 |
| **average±sd** | 0.796±0.045 | 0.762±0.049 |  |  |

sd = standard deviation

**Supplementary Table 8:** ANOSIM results to test yearly differences in the euphotic and aphotic zones. ASV data were CLR transformed with an Aitchison distance matrix.

|  | **Euphotic** | **Aphotic** |  |  |
| --- | --- | --- | --- | --- |
| **Global test** | 0.091 | 0.276 |  |  |
| **Significance** | 0.001 | 0.001 |  |  |
| **Permutations** | 999 | 999 |  |  |
|  |  |  |  |  |
|  | **R-value** | |  |  |
| **Groups** | **Euphotic** | **Aphotic** | **Significance** | **Permutations** |
| 2014-2015 | 0.089 | 0.224 | 0.001 | 999 |
| 2014-2016 | 0.155 | 0.427 | 0.001 | 999 |
| 2014-2017 | 0.11 | 0.231 | 0.001 | 999 |
| 2015-2016 | 0.074 | 0.302 | 0.001 | 999 |
| 2015-2017 | 0.069 | 0.238 | 0.001 | 999 |
| 2016-2017 | 0.059 | 0.256 | 0.001 | 999 |

**Supplementary Table 9:** Indicator analysis for samples in the euphotic zone. ASV data were conglomerated at a phylum level and CLR transformed prior to indicator analysis using the ‘Indicspecies’ package (De Cáceres, 2013).

| euphotic zone | |  |  |  |
| --- | --- | --- | --- | --- |
| Season | **Month** | **Phyla** | **Pearson’s phi coefficient** | **p-value** |
| Spring | **Apr** | Synergistota | 0.3 | 0.0001 |
|  |  | Proteobacteria | 0.247 | 0.0001 |
|  |  | Cloacimonadota | 0.221 | 0.0001 |
|  |  | Armatimonadota | 0.213 | 0.0001 |
|  | **May** | Modulibacteria | 0.299 | 0.0001 |
|  |  | Gemmatimonadota | 0.257 | 0.0001 |
|  |  |  |  |  |
| Summer | **Jun** | Deinococcota | 0.289 | 0.0001 |
|  |  | Fermentibacterota | 0.287 | 0.0001 |
|  |  | Bacteroidota | 0.245 | 0.0001 |
|  |  | Thermotogota | 0.227 | 0.0001 |
|  |  | Chloroflexi | 0.224 | 0.0001 |
|  |  | Actinobacteriota | 0.211 | 0.0001 |
|  |  | Calditrichota | 0.187 | 0.0004 |
|  |  | Dependentiae | 0.128 | 0.0401 |
|  | **Aug** | Verrucomicrobiota | 0.235 | 0.0001 |
|  |  |  |  |  |
| Autumn | **Sep** | Cyanobacteria | 0.401 | 0.0001 |
|  |  | Campylobacterota | 0.239 | 0.0001 |
|  |  | Acidobacteriota | 0.195 | 0.0002 |
|  |  | Fibrobacterota | 0.147 | 0.0069 |
|  | **Nov** | Bdellovibrionota | 0.212 | 0.0001 |
|  |  |  |  |  |
| Winter | **Dec** | Planctomycetota | 0.266 | 0.0001 |
|  |  | Myxococcota | 0.237 | 0.0001 |
|  |  | Hydrogenedentes | 0.221 | 0.0001 |
|  | **Jan** | Nitrospinota | 0.414 | 0.0001 |
|  |  | Fusobacteriota | 0.288 | 0.0001 |
|  |  | Firmicutes | 0.244 | 0.0001 |
|  |  | Desulfobacterota | 0.188 | 0.0004 |

**Note:** Missing months did not show any ‘indicator’ phyla.

**Supplementary Table 10:** Indicator analysis for samples in the aphotic zone. ASV data were conglomerated at a phylum level and CLR transformed prior to indicator analysis using the ‘Indicspecies’ package (De Cáceres, 2013).

| aphotic zone | |  |  |  |
| --- | --- | --- | --- | --- |
| Season | **Month** | **Phyla** | **Pearson’s phi coefficient** | **p-value** |
| Spring | **Mar** | Proteobacteria | 0.258 | 0.0053 |
|  | **Apr** | Synergistota | 0.349 | 0.0001 |
|  |  | Bacteroidota | 0.34 | 0.0001 |
|  |  | Armatimonadota | 0.319 | 0.0002 |
|  |  | Cloacimonadota | 0.319 | 0.0002 |
|  |  | Thermotogota | 0.319 | 0.0002 |
|  |  | Gemmatimonadota | 0.319 | 0.0002 |
|  |  | Deinococcota | 0.27 | 0.0049 |
|  | **May** | Verrucomicrobiota | 0.387 | 0.0001 |
|  |  | Fermentibacterota | 0.345 | 0.0011 |
|  |  | Modulibacteria | 0.345 | 0.0011 |
|  |  |  |  |  |
| Summer | **Jun** | Dependentiae | 0.269 | 0.0083 |
|  |  |  |  |  |
| Autumn | **Oct** | Planctomycetota | 0.252 | 0.0005 |
|  |  | Cyanobacteria | 0.217 | 0.0105 |
|  |  | Hydrogenedentes | 0.214 | 0.0062 |
|  | **Nov** | Nitrospinota | 0.303 | 0.0001 |
|  |  |  |  |  |
| Winter | **Dec** | Desulfobacterota | 0.183 | 0.0186 |
|  | **Jan** | Fusobacteriota | 0.26 | 0.0079 |
|  |  | Firmicutes | 0.221 | 0.0272 |
|  | **Feb** | Myxococcota | 0.247 | 0.0039 |

**Note:** Missing months did not show any ‘indicator’ phyla.

**Supplementary Table 11:** Inferred function indicator analysis for samples in the euphotic zone. MetaCyc data CLR transformed prior to indicator analysis using the ‘Indicspecies’ package (De Cáceres, 2013).

| Season | Month | Pathways | Pearson’s phi coefficient | p-value |
| --- | --- | --- | --- | --- |
| Spring | **Mar** | Propionate.Degradation | 0.209 | 1.00E-04 |
|  | **Apr** | Lipid.Biosynthesis | 0.339 | 1.00E-04 |
|  |  | Vitamin.Biosynthesis | 0.252 | 1.00E-04 |
|  |  | Carbohydrates.Degradation | 0.194 | 1.00E-04 |
|  | **May** | Nitroaromatic.Degradation | 0.294 | 1.00E-04 |
|  |  | Fatty.Acid.and.Lipid.Degradation | 0.281 | 1.00E-04 |
|  |  | Phosphorus.Compounds | 0.238 | 1.00E-04 |
|  |  | Sugar.Nucleotides.Biosynthesis | 0.234 | 1.00E-04 |
|  |  |  |  |  |
| Summer | **Jun** | Aromatic.compound.degradation | 0.289 | 0.0001 |
|  |  | Purine.Degradation | 0.276 | 0.0001 |
|  |  | Degradation | 0.256 | 0.0001 |
|  |  | Nitrogen.Degradation | 0.149 | 0.002 |
|  |  | Siderophores.Biosynthesis | 0.131 | 0.0047 |
|  | **Jul** | Aromatic.Compounds.Biosynthesis | 0.34 | 1.00E-04 |
|  |  | Nucleotide.Biosynthesis | 0.314 | 1.00E-04 |
|  |  | Cell.Structure.Cell.Wall.Biosynthesis | 0.312 | 1.00E-04 |
|  |  | Secondary.Metabolite.Biosynthesis | 0.299 | 1.00E-04 |
|  |  | Amino.Acid.Biosynthesis | 0.291 | 1.00E-04 |
|  |  | Sugars.And.Acids.Degradation | 0.281 | 1.00E-04 |
|  |  | Energy.Metabolism | 0.276 | 1.00E-04 |
|  |  | Phospholipid.Biosynthesis | 0.247 | 1.00E-04 |
|  |  | Carbohydrates.Biosynthesis | 0.244 | 1.00E-04 |
|  |  | Sulfur.Metabolism | 0.235 | 1.00E-04 |
|  |  | Fermentation | 0.227 | 1.00E-04 |
|  |  | Alcohol.Degradation | 0.122 | 1.00E-04 |
|  | **Aug** | Isopentenyl.Diphosphate.Biosynthesis | 0.287 | 1.00E-04 |
|  |  | Cofactor.Biosynthesis | 0.282 | 1.00E-04 |
|  |  | Sugars.And.Polysaccharides.Degradation | 0.249 | 1.00E-04 |
|  |  | Polyamine.Biosynthesis | 0.167 | 7.00E-04 |
|  |  |  |  |  |
| Autumn | **Sep** | Chlorophyll.Biosynthesis | 0.34 | 1.00E-04 |
|  |  | CO2.Fixation | 0.266 | 1.00E-04 |
|  |  | Glycan.Pathways | 0.23 | 1.00E-04 |
|  |  | Nitrate.Reduction | 0.168 | 3.00E-04 |
|  |  |  |  |  |
| Winter | **Nov** | Formaldehyde.Assimilation | 0.159 | 1.00E-04 |
|  | **Dec** | Mevalonate.Pathways | 0.256 | 1.00E-04 |
|  | **Jan** | Methanogenesis | 0.333 | 1.00E-04 |
|  |  | Denitrification | 0.242 | 1.00E-04 |

**Note:** Missing months did not show any ‘indicator’ pathways.

**Supplementary Table 12:** Inferred function indicator analysis for samples in the aphotic zone. MetaCyc data CLR transformed prior to indicator analysis using the ‘Indicspecies’ package (De Cáceres, 2013).

| Season | Month | Pathways | Pearson’s phi coefficient | p-value |
| --- | --- | --- | --- | --- |
| Spring | **Mar** | Propionate.Degradation | 0.154 | 4.00E-04 |
|  | **Apr** | Lipid.Biosynthesis | 0.283 | 1.00E-04 |
|  |  | Purine.Degradation | 0.197 | 1.00E-04 |
|  |  | Vitamin.Biosynthesis | 0.185 | 1.00E-04 |
|  |  | Polyamine.Biosynthesis | 0.165 | 1.00E-04 |
|  |  | Carbohydrates.Degradation | 0.163 | 3.00E-04 |
|  | **May** | Nitroaromatic.Degradation | 0.266 | 1.00E-04 |
|  |  | Fatty.Acid.and.Lipid.Degradation | 0.231 | 1.00E-04 |
|  |  | Phosphorus.Compounds | 0.175 | 1.00E-04 |
|  |  |  |  |  |
| Summer | **Jun** | Aromatic.compound.degradation | 0.212 | 0.0001 |
|  |  | Degradation | 0.151 | 0.0001 |
|  |  | Siderophores.Biosynthesis | 0.111 | 0.0066 |
|  | **Jul** | Sugars.And.Acids.Degradation | 0.24 | 0.0001 |
|  |  | Aromatic.Compounds.Biosynthesis | 0.238 | 0.0001 |
|  |  | Cell.Structure.Cell.Wall.Biosynthesis | 0.237 | 0.0001 |
|  |  | Nucleotide.Biosynthesis | 0.212 | 0.0001 |
|  |  | Amino.Acid.Biosynthesis | 0.197 | 0.0001 |
|  |  | Secondary.Metabolite.Biosynthesis | 0.193 | 0.0001 |
|  |  | Energy.Metabolism | 0.183 | 0.0001 |
|  |  | Sulfur.Metabolism | 0.148 | 0.0012 |
|  |  | Alcohol.Degradation | 0.117 | 0.0001 |
|  |  | Carbohydrates.Biosynthesis | 0.107 | 0.025 |
|  | **Aug** | Isopentenyl.Diphosphate.Biosynthesis | 0.231 | 1.00E-04 |
|  |  | Cofactor.Biosynthesis | 0.213 | 1.00E-04 |
|  |  | Phospholipid.Biosynthesis | 0.187 | 1.00E-04 |
|  |  | Sugars.And.Polysaccharides.Degradation | 0.176 | 2.00E-04 |
|  |  | Fermentation | 0.132 | 7.00E-03 |
|  |  |  |  |  |
| Autumn | **Sep** | Chlorophyll.Biosynthesis | 0.268 | 1.00E-04 |
|  |  | Glycan.Pathways | 0.169 | 2.00E-04 |
|  |  | Nitrate.Reduction | 0.126 | 2.00E-03 |
|  | **Oct** | CO2.Fixation | 0.171 | 2.00E-04 |
|  |  |  |  |  |
| Winter | **Jan** | Methanogenesis | 0.231 | 1.00E-04 |
|  |  | Denitrification | 0.217 | 1.00E-04 |
|  |  | Mevalonate.Pathways | 0.196 | 1.00E-04 |
|  | **Feb** | Formaldehyde.Assimilation | 0.137 | 1.00E-04 |

**Note:** Missing months did not show any ‘indicator’ pathways.

**References:**

De Cáceres, M. (2013). How to use the indicspecies package (ver. 1.7. 1). *R Proj*, *29*.

Oksanen, J., Kindt, R., Legendre, P., O’Hara, B., Stevens, M. H. H., Oksanen, M. J., & Suggests, M. (2007). The vegan package. *Community Ecology Package*, *10*(631–637), 719.
